# Linking DNA repair and cell cycle progression through serine ADP-ribosylation of histones

**DOI:** 10.1038/s41467-021-27867-4

**Published:** 2022-01-13

**Authors:** Julien Brustel, Tetsuya Muramoto, Kazuki Fumimoto, Jessica Ellins, Catherine J. Pears, Nicholas D. Lakin

**Affiliations:** 1 Department of Biochemistry, University of Oxford, South Parks Road, Oxford, UK; 2 Department of Biology, Faculty of Science, Toho University, Funabashi, Chiba Japan

**Keywords:** Double-strand DNA breaks, PolyADP-ribosylation

## Abstract

Although serine ADP-ribosylation (Ser-ADPr) by Poly(ADP-ribose)-polymerases is a cornerstone of the DNA damage response, how this regulates DNA repair and genome stability is unknown. Here, we exploit the ability to manipulate histone genes in *Dictyostelium* to identify that ADPr of the histone variant H3b at S10 and S28 maintains genome stability by integrating double strand break (DSB) repair with mitotic entry. Given the critical requirement for mitotic H3S10/28 phosphorylation, we develop separation of function mutations that maintain S10 phosphorylation whilst disrupting ADPr. Mechanistically, this reveals a requirement for H3bS10/28 ADPr in non-homologous end-joining by recruiting Ku to DSBs. Moreover, this also identifies H3bS10/S28 ADPr is critical to prevent premature mitotic entry with unresolved DNA damage, thus maintaining genome stability. Together, these data demonstrate how serine ADPr of histones coordinates DNA repair with cell cycle progression to maintain genome stability.

## Abstract (web summary)

Poly(ADP-ribose)-polymerases (PARPs) are a cornerstone of the DNA damage response that promote DNA repair by modifying target proteins with ADP-ribose. Here, the authors show serine ADP-ribosylation of the H3 variant H3b maintains genome stability by coupling DNA repair with mitotic entry in *Dictyostelium* by regulating double strand break repair by nonhomologous end-joining (NHEJ).

## Introduction

Poly(ADP-ribose) polymerases (PARPs) catalyse the NAD^+^-dependent addition of single ADP-ribose moieties or chains onto target proteins by mono- or poly-ADP-ribosylation, respectively. ADP-ribosylation (ADPr) has been implicated in a wide variety of cellular processes including cell growth and differentiation, transcriptional regulation and programmed cell death^1^. However, the best established role of this modification is in maintaining genome integrity through DNA repair^2^.

Of the 17 genes that contain PARP catalytic domains several have been implicated in the DNA damage response, including PARP1 and PARP2 that catalyse poly-ADPr, and PARP3 that performs mono-ADPr^3^. PARPs are critical for the repair of DNA strand breaks by ADP-ribosylating factors at DNA lesions to promote the recruitment of chromatin remodelling and repair factors through PAR-interaction domains present in these proteins. For example, PARP1 and PARP2 are activated upon binding to SSBs and through ADPr of target proteins at the break, promote XRCC1 and ALC1 recruitment to damage sites to regulate the assembly and turnover of additional factors that facilitate DNA repair^4–10^. In contrast, PARP3 responds to DNA DSBs, promoting the assembly of non-homologous end-joining (NHEJ) factors at DNA lesions^11,12^. Whilst PARP1 has also been implicated in remodelling chromatin at DSBs to promote NHEJ^13^, it is also required for alternative-NHEJ, a pathway that employs micro-homology-based repair to resolve DSBs in the absence of conventional NHEJ^14^. PARP1 and PARP2 also regulate replication-associated mechanisms including Okazaki fragment processing^15^ and replication-associated repair by promoting Mre11 recruitment to stalled/damaged replication forks^16–18^, maintaining regressed forks by inhibiting the RECQ1 helicase^19^, and stabilising homologous recombination (HR) factors at these structures^5^.

However, whilst the pathways that PARPs function in to maintain genome integrity are becoming increasingly well-defined, the mechanistic basis of this regulation is less clear. ADPr of nuclear proteins, most notably histones, has been known for many years^20^. However, it is not until recently that advances in mass spectrometry have provided a detailed map of the ADP-ribosylome^21–25^. Glutamate (Glu; D) and aspartate (Asp; E) were initially identified as key ADP-ribose acceptors and site-specific ADPr of these amino acids has been implicated in DNA repair and cell type specification^26–29^. However, ADPr of Glu and Asp are relatively low abundance events following genotoxic stress. Instead, a key advance in our understanding was the identification of histone PARylation factor 1 (HPF1), a PARP1/PARP2 interacting protein that directs ADPr of histones and other target proteins on serine^24,30^. Serine is the major acceptor for ADP-ribose in response to DNA damage, and all core histones are ADPr in response to genotoxic stress, predominantly in the context of a KS motif^23,31^. Given the high density of post-translational modifications (PTMs) within histone tails, serine ADPr (Ser-ADPr) can impact on modification of other sites within histones such as lysine acetylation^32,33^. Indeed, Ser-ADPr itself may block the ability to phosphorylate these amino acids and vice versa^34^, suggesting these PTMs may have opposing roles in regulating variety processes. However, the functional significance of these relationships is unknown.

This lack of mechanistic insight is due, in part, to the absence of an appropriate experimental platform to assess the role of site-specific histone ADPr events in vivo. Multiple copies of core histone genes in vertebrates make the manipulation of specific PTM sites at endogenous histone loci challenging. This is exacerbated by the absence of PARPs in commonly used model organisms to study DNA repair where this technology is available, precluding an analysis of histone ADPr in these systems. In this context, the amoeba *Dictyostelium discoidium* is an ideal model organism to study histone ADPr in DNA repair and genome stability. We and others identified a number of vertebrate DNA repair components in *Dictyostelium* that are lost or show limited conservation in other model organisms used to study the DNA damage response (DDR)^35–39^. This is particularly striking with PARPs and the mechanistic basis of how these enzymes regulate DNA DSB repair is conserved with vertebrates^35,40–43^. *Dictyostelium* is also ideally suited to study how site-specific modification of histones regulates a variety of processes. It contains a wider variety of histone variants that are more similar to vertebrates than other simple eukaryotic model organisms^44–46^. The major PTMs on histones are also observed in this organism, including ADPr^45–49^. Importantly, *Dictyostelium* also contains single copy histone genes that are amenable to genetic manipulation, opening up the possibility to perform gene replacement and site-specific mutation strategies to assess the functional significance of histone PTMs^45,50,51^.

Recently, we exploited these unique characteristics of *Dictyostelium* to develop this organism as a model to identify site-specific ADPr events and characterise how they regulate DNA repair and genome stability^43,50^. Here we build on these studies, using this system to identify that serine ADPr (Ser-ADPr) is conserved in *Dictyostelium* and to assess how Ser-ADPr of histones coordinates DNA repair and mitotic entry following genotoxic stress to maintain genome stability.

## Results

### The histone variant H3b is required to maintain genome stability through DNA DSB repair

**Fig. 1:**
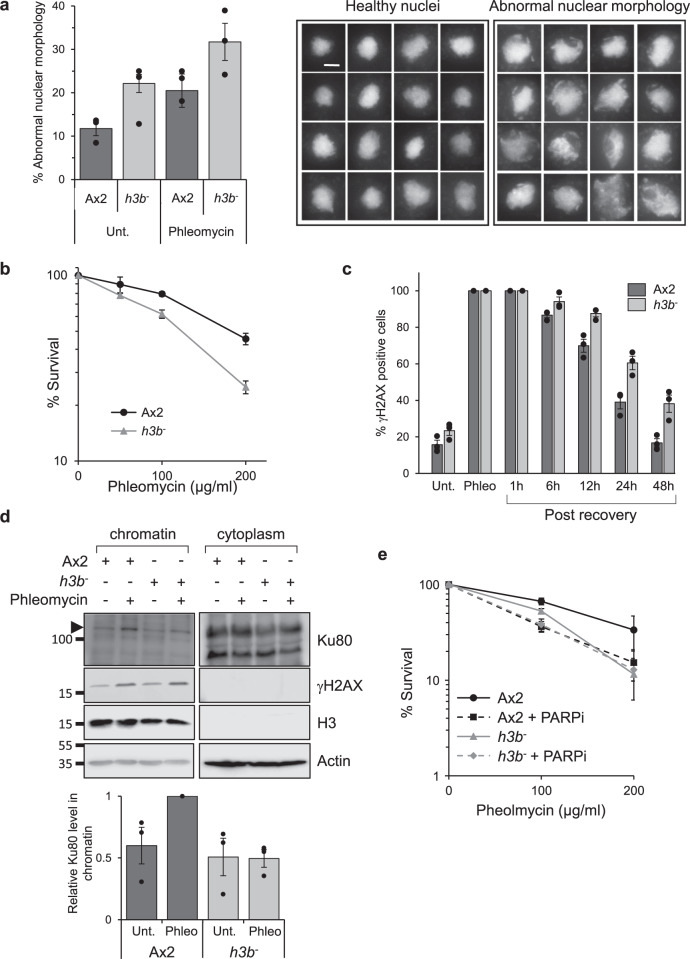
H3b is required for DSB repair and genome stability. **a** Abnormal nuclear morphology in the *h3b*^*−*^ strain. Left panel: Quantification of cells displaying abnormal nuclear morphology in Ax2 or *h3b*^*−*^ cells either in control conditions or 48 h after a 1 h exposure to phleomycin (*n* = 3; individual data points are shown, error bars represent the SEM). Right panel: representative pictures of healthy and abnormal nuclei. DNA is stained with DAPI and each nucleus has a diameter of 14.5 μm. Scale bar represents 5 μm. **b** Ax2 or *h3b*^*−*^ cells were exposed to phleomycin for 1 h at the indicated concentrations and cell survival assessed by clonogenic survival assays (data represent three biological repeats, error bars represent the SEM). **c** Ax2 or *h3b*^*−*^ cells were treated for 1 h with phleomycin and following recovery in fresh media, cells with >5 γH2AX foci assessed by immunofluorescence. (data represent three biological repeats; individual data points are shown, error bars represent the SEM). **d** Following exposure of Ax2 or *h3b*^*−*^ cells to phleomycin for 1 h, cytoplasmic and chromatin fractions were prepared from cells and western blotting performed using the indicated antibodies (left panel). Enrichment of Ku80 in chromatin fractions was quantified from three independent experiments (right panel). Molecular weight markers are indicated in kDa. **e** Ax2 or *h3b*^*−*^ cells were exposed to phleomycin for 1 h at the indicated concentrations either the absence or presence of olaparib (PARPi) and cell survival assessed by clonogenic survival assays (data represent four biological repeats; error bars represent the SEM). Source data are provided in the Source Data file.

Given the ability to manipulate histone genes in *Dictyostelium* and that histone H3 is a major acceptor of ADP-ribose in response to DNA damage in vertebrates^23,31,52^, we assessed the impact of manipulating the two major *Dictyostelium* H3 variant genes on genome stability and DNA repair. Despite repeated attempts no strains with disruption of the *h3a* gene have been generated, suggesting an essential requirement for this histone variant in *Dictyostelium* cells. In contrast, an *h3b* null strain has been successfully generated (*h3b*^*−*^)^45^ and strikingly, these cells exhibit elevated levels of abnormal nuclear morphology relative to parental Ax2 cells (Fig. 1a). More than 80% of these nuclei exhibit γH2AX staining (Supplementary Fig. 1A), suggesting they represent cells with increased genome instability and/or DNA damage. Abnormal nuclear morphology is elevated in untreated *h3b*^*−*^ relative to Ax2, rather than dramatically increasing in response to phleomycin, suggesting genome instability may be a consequence of spontaneous or endogenous DNA damage (Fig. 1a, left panel). Nevertheless, the *h3b*^*−*^ strain is more sensitive to phleomycin relative to Ax2 cells (Fig. 1b), suggesting an inability of these cells to repair DNA strand breaks. Additionally, whilst induction and decay rates of γH2AX are similar in Ax2 and *h3b*^*−*^ cells following a transient exposure to phleomycin (e.g. Fig. 1c, d and Supplementary Fig. 1B), recovery is delayed in the *h3b*^*−*^ strain, further supporting a requirement for H3b in initiating repair of DNA damage.

Given the striking conservation of vertebrate DSB components in *Dictyostelium*^36–39^, we considered whether these pathways are compromised in the *h3b*^*−*^ strain. Strains defective in the key HR gene *exonuclease I* (*exo1*^*−*^) are sensitive to DNA DSBs. However, *h3b*^*−*^ cells show radiosensitivity between Ax2 and *exo1*^*−*^ cells, similar to that observed for a NHEJ-defective strain (*ku80*^*−*^; Supplementary Fig. 2A), suggesting disruption of *h3b* results in defective NHEJ rather than HR. Recruitment of repair factors to DSBs through PAR-binding motifs is a key step in initiating NHEJ in vertebrates^11^ and *Dictyostelium*^35^. Therefore, we also considered whether H3b is required for assembly of NHEJ and/or HR factors in chromatin following DSBs. Whilst we see robust enrichment of Ku80 in chromatin in response to DSBs in Ax2 cells, this is reduced in the *h3b*^*−*^ strain (Fig. 1d). In contrast, assembly of Rad51 in chromatin following DSBs is similar in Ax2 and *h3b*^*−*^ cells, supporting the hypothesis that HR is not dependent on H3b (Supplementary Fig. 2B). Taken together, these data indicate that whilst loss of H3b has little impact on markers of HR, it results in defective DNA repair that is reflected in a reduced ability to assemble NHEJ factors such as Ku80 at sites of DNA damage.

### Histone H3b is ADPr on serines in response to DNA DSBs

Our previous work identified that disruption of the *Dictyostelium* PARP Adprt1a results in radiosensitivity and an inability to recruit the Ku heterodimer to DNA DSBs via a PAR-interaction domain in Ku70^35,40^. Given the similarity of these phenotypes with *h3b*^*−*^ cells (Fig. 1), we considered the possibility that these events are regulated through ADPr of H3b. Consistent with this hypothesis, whilst PARP inhibitors (PARPi) sensitise parental Ax2 cells to DSBs induced by phleomycin, they do not further sensitise *h3b*^*−*^ cells to DNA damage, indicating that PARPs and H3b likely function in the same pathway with regards radioresistance (Fig. 1e and Supplementary Fig. 2C).

**Fig. 2:**
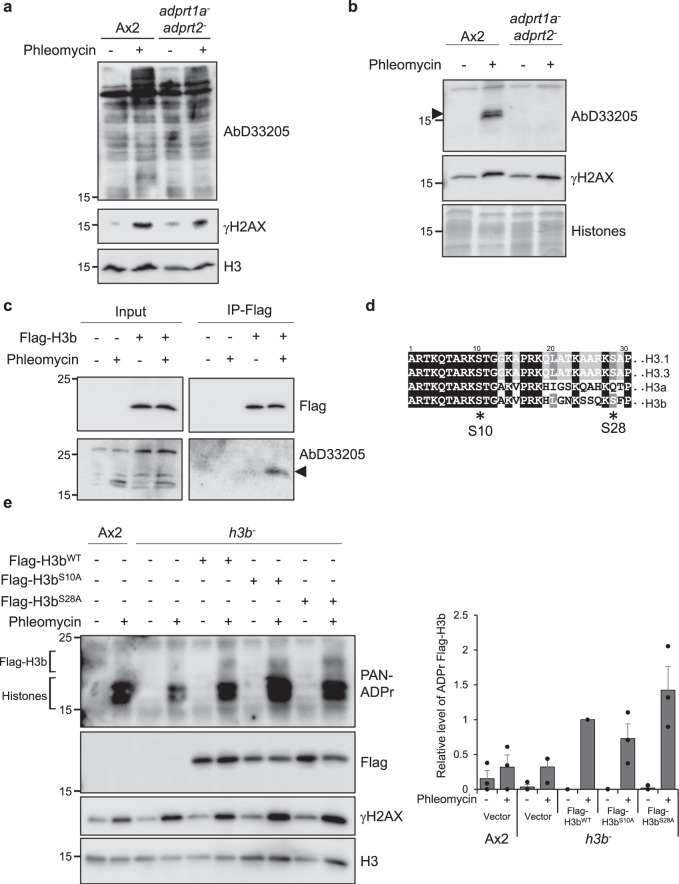
H3b is ADP ribosylated on serine in response to DNA damage. **a**, **b** Ax2 or *adprt1a*^*−*^*adprt2*^*−*^ cells were treated with phleomycin and whole cell (a) or acid (**b**) extracts western blotted with antibodies as indicated. Molecular weight markers are indicated in kDa. Representative pictures of three biological repeats are presented. **c** *h3b*^*−*^ cells containing empty vector, or expressing Flag-H3b^wt^, were left untreated or exposed to phleomycin. Following preparation of denatured chromatin, Flag immunoprecipitation was performed. Input extracts or immunoprecipitates were subjected to Western blotting using the indicated antibodies. Molecular weight markers are indicated in kDa. Representative pictures of at least three independent experiments. **d** Sequence comparison of human histone H3.1 and H3.3 with the *Dictyostelium* H3 variants H3a and H3b. S10 and S28, the main ADP-ribosylation targets in vertebrates are indicated. **e** Ax2 or *h3b*^*−*^ cells containing empty vector, or expressing Flag-H3b^WT^, Flag-H3b^S10A^ or Flag-H3b^S28A^ were left untreated or exposed to phleomycin. Following preparation of acid extracts, western blotting was performed using the indicated antibodies. ADP-ribosylated endogenous histones and Flag-H3b are highlighted. Enrichment of Flag-H3b in chromatin fractions was quantified (right panel; *n* = 3; individual data points are shown and error bars represent the SEM). Molecular weight markers are indicated in kDa. Source data are provided in the Source Data file.

To further assess the link between ADPr of H3b and DNA repair, we next assessed whether this histone variant is ADPr in response to DSBs. Vertebrate histones, including H3, are ADPr on serine in response to genotoxic stress^23^. Therefore, we investigated whether Ser-ADPr similarly occurs in *Dictyostelium*. We detect Adprt1a/Adprt2-dependent ADPr in *Dictyostelium* whole-cell extracts using an antibody that recognises HPF1-dependent ADPr events in vivo and therefore represents largely Ser-ADPr (D33205; Fig. 2a)^34^. A variety of bands that are recognised by D33205 are induced upon DNA damage, suggesting serine may be ADP-ribosylated on a variety of substrates in response to DSBs. Several lower molecular weight proteins are recognised by D33205 and consistent with these protein species representing histones, they co-purify with basic proteins during acid extraction, a protocol that enriches for histone proteins^45^ (Fig. 2b). To more directly investigate whether H3b is a target for Ser-ADPr, we expressed Flag-tagged H3b (Flag-H3b^wt^) in the *h3b*^*−*^ strain. Flag-H3b immunoprecipitated from cells is recognised by D33205 and this signal is induced upon induction of DNA damage by phleomycin (Fig. 2c). Taken together, these data indicate that Ser-ADPr is conserved in *Dictyostelium* and that histone H3b is a target for this PTM in response to DNA damage.

Next, we assessed which sites on H3b are ADPr in response to DSBs. Studies in vertebrates reveal that serines 10 (S10) and 28 (S28) are the main ADPr acceptors on histone H3 in response to DNA damage^23,31,52^. S10 and S28 are conserved in *Dictyostelium* H3b, whilst H3a is conserved only at S10 (Fig. 2d). To assess whether H3b S10 and S28 are ADP-ribosylated, we expressed Flag-tagged H3b bearing mutations at S10 (Flag-H3b^S10A^) or S28 (Flag-H3b^S28A^) in *h3b*^*−*^ cells and assessed ADPr following DNA damage by western blotting of histone-enriched acid extracts with a reagent that recognises poly- and mono-ADPr proteins. Consistent with ADPr of recombinant H3b, a higher molecular weight DNA damage-induced species is apparent specifically in Flag-H3b^WT^ expressing cells relative to non-expressing Ax2 cells (Fig. 2e). This ADPr species is also apparent in cells expressing either Flag-H3b^S10A^ or Flag-H3b^S28A^, indicating that mutation of these amino acids alone does not significantly impact on the ability of H3b to be ADP-ribosylated in response to DNA DSBs. Together, these data indicate that whilst H3b is robustly ADP-ribosylated in response to DNA DSBs, either S10/S28 are not modified, or that similar to vertebrates^31^ these events are not exclusive to either of these amino acids.

### Mutations that maintain phosphorylation whilst disrupting Ser-ADPr reveal H3b S10 and S28 are ADPr in response to DNA DSBs

Given that disruption of S10 or S28 does not disrupt H3b ADPr, we attempted to mutate these sites in combination. However, despite repeated attempts, we were unable to express Flag-H3b^S10AS28A^ in the *h3b*^*−*^ strain, suggesting that expression of this mutant is toxic to cells. Phosphorylation of S10 and S28 by Aurora A and Aurora B is a common marker of mitotic entry and these modifications are critical for faithful mitotic progression in a variety of organisms^53^. Given phosphorylation of *Dictyostelium* H3 at S10 is detected by mass spectrometry^46^, we considered the possibility that toxicity of the Flag-H3b^S10AS28A^ mutant is a consequence of deregulated cell cycle progression due to defective H3 phosphorylation during mitosis. We therefore developed a mutation strategy to separate the function of H3b ADPr and phosphorylation, allowing us to establish the biological significance of H3 ADPr without impacting on its phosphorylation status during the cell cycle.

**Fig. 3:**
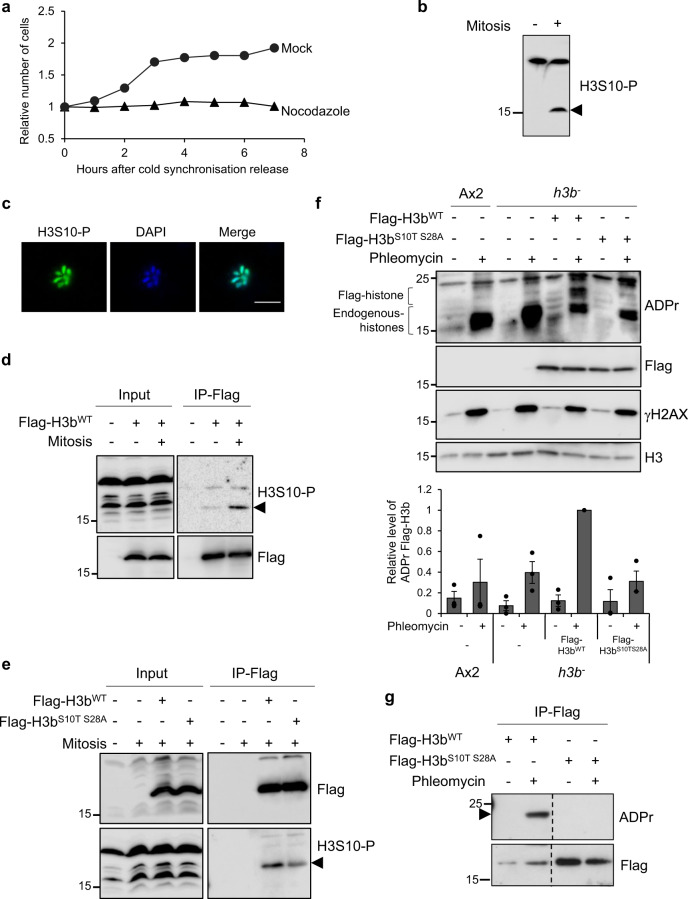
Separation of function of H3b serine phosphorylation and ADP-ribosylation. **a** Ax2 cells were blocked in G2 and then released from cell cycle arrest in the absence or presence of nocodazole as indicated. Progression through mitosis was monitored by assessing cell numbers at times following release from G2. Quantification of one representative experiment from 3 biological repeats. **b**, **c** Asynchronous or nocodazole arrested Ax2 cells were subjected to Western blotting of whole cell extracts (**b**) or immunofluorescence (**c**) using an H3S10-P specific antibody. Molecular weight markers are indicated in kDa. Scale bar 5 μm. **d**, **e** Cells containing empty vector or expressing Flag-H3b^WT^ (**d**) and Flag-H3b^WT^ or Flag-H3b^S10TS28A^ (**e**) were left untreated or synchronised in mitosis with a nocodazole block as indicated. Input or Flag-immunoprecipitates were subjected to Western blotting using the indicated antibodies. Molecular weight markers are indicated in kDa. **f** The indicated cell lines were left untreated or exposed to phleomycin. Western blotting of acid extracts was performed using the indicated antibodies. ADP-ribosylated endogenous histones and Flag-H3b are highlighted. Enrichment of Flag-H3b in chromatin fractions was quantified (lower panel; *n* = 3; individual data points are shown and error bars represent the SEM). Molecular weight markers are indicated in kDa. **g** *h3b*^*−*^ cells expressing Flag-H3b^WT^ or Flag-H3b^S10TS28A^ were left untreated or exposed to phleomycin as indicated. Western blotting of Flag-immunoprecipitates was performed using the indicated antibodies. Molecular weight markers are indicated in kDa. All experiments represent at least three biological repeats. Source data are provided in the Source Data file.

To address this question, we initially developed an assay to assess H3 phosphorylation and ADPr during mitosis. Cells were synchronised in the G2 phase of the cell cycle using standard procedures^40^ and following release from this block, progression through mitosis assessed by monitoring synchronous cell doubling (Fig. 3a). Consistent with H3 S10 phosphorylation during mitosis, arresting these cells at anaphase by release into nocodozole results in decoration of mitotic chromosomes with a H3S10-P phospho-specific antibody and induction of H3 phosphorylation during mitosis (Fig. 3b, c). To specifically assess the phosphorylation status of H3b during mitosis, Flag-H3b^WT^ was immunoprecipitated from asynchronous or mitotic arrested cells. Consistent with phosphorylation of H3b at S10 during mitosis, Flag-H3b^WT^ is recognised by the H3S10-P antibody and this is induced in mitotic arrested cells (Fig. 3d).

Whilst serine is the main target for DNA damage-induced ADPr, threonine is not ADP-ribosylated by PARP1/HPF1 in vitro^32^ and has not been detected by MS in cells^23,24^. We therefore hypothesised that a S10T mutation will maintain phosphorylation at this site, whilst disrupting ADPr. Consistent with this idea, in contrast to expression of Flag-H3b^S10AS28A^, *h3b*^*−*^ cells expressing Flag-H3b^S10TS28A^ are viable and exhibit similar growth rates to *h3b*^*−*^ cells expressing wild-type Flag-H3b (Supplementary Fig. 3A), indicating this mutation combination is not toxic to cells. Moreover, Flag-H3b^S10TS28A^ immunoprecipitated from mitotic extracts is recognised by the H3S10-P antibody, indicating this mutant is phospho-competent during mitosis (Fig. 3e). Next, we assessed the ADPr status of H3b^S10T^, H3b^S28A^ and Flag-H3b^S10TS28A^ in response to DSBs. Whilst the S10T or S28A mutations reduce ADPr of H3b, it is still evident, indicating both these residues are targeted independently of each other in response to DNA damage (Supplementary Fig. 4A). However, whilst DNA damage-induced ADPr of Flag-H3b^WT^ is readily detectable in histone-enriched extracts or immunoprecipitates, we were unable to detect ADPr of Flag-H3b^S10TS28A^ (Fig. 3f, g). Additionally, whilst Flag-H3b co-purified with ADP-ribosylated proteins affinity purified from phleomycin treated cells, Flag-H3b^S10TS28A^ did not (Supplementary Fig. 3B). Taken together these data indicate S10 and S28 of H3b are ADP-ribosylated in response to DNA DSBs. Critically, they also indicate that in the context of H3bS10, generating S to T mutants disrupts ADPr whilst maintaining phosphorylation, offering the opportunity to separate the function of phosphorylation and ADPr in a variety of processes.

### ADPr of H3bS10/S28 is necessary for efficient DSBs repair via NHEJ

**Fig. 4:**
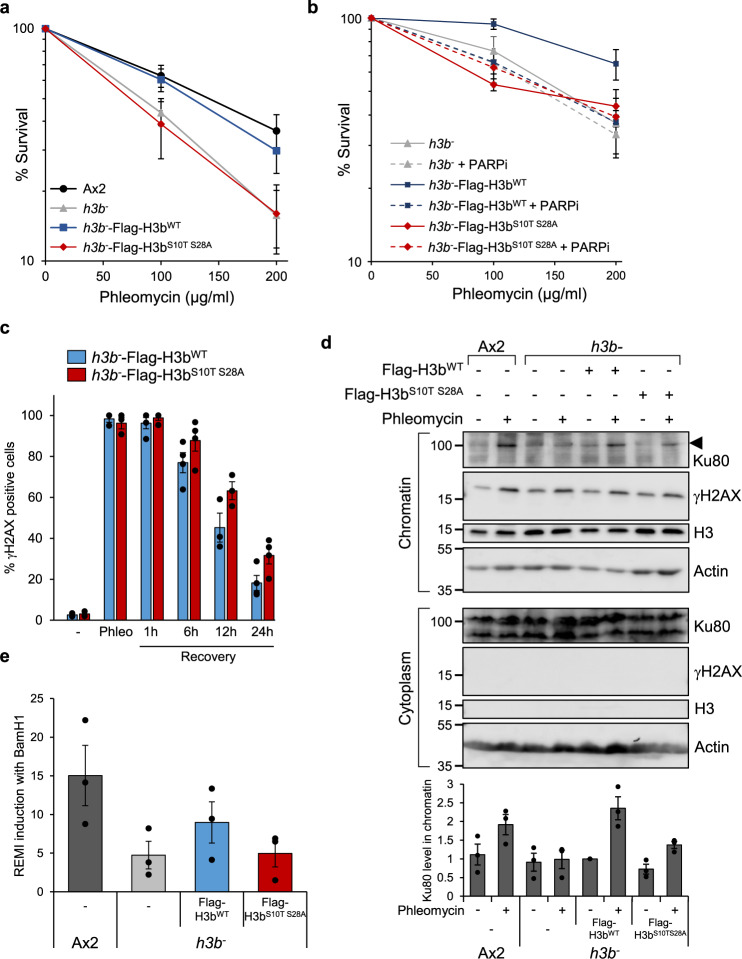
H3b ADP-ribosylation is required for DSB repair. **a** Ax2 cells, or *h3b*^*−*^ cells expressing Flag-H3b^WT^ or Flag-H3b^S10T S28A^ were exposed to phleomycin at the indicated concentrations and cell viability assessed by clonogenic survival assays (data represent seven biological repeats; error bars represent the SEM). **b** *h3b*^*−*^ cells alone, or expressing Flag-H3b^WT^ or Flag-H3b^S10T S28A^ were exposed to phleomycin in the absence or presence of PARP inhibitors (olaparib; PARPi) as indicated and cell viability assessed by clonogenic survival assays (data represent three biological repeats; error bars represent the SEM). **c** *h3b*^*−*^ cells expressing Flag-H3b^WT^ or Flag-H3b^S10T S28A^ were left untreated (−) or exposed to phleomycin for 1 h (Phleo). Following removal of phleomycin, recovery of cells was analysed at the time points indicated. DNA damage was assessed by scoring γH2AX nuclei with >5 foci (*n* = 3; individual data points are shown and error bars represent the SEM). **d** Ax2, *h3b*^*−*^ cells, or *h3b*^*−*^ cells expressing Flag-H3b^WT^ or Flag-H3b^S10T S28A^ were left untreated or exposed to phleomycin as indicated. Cytoplasmic and chromatin fractions were prepared from cells and western blotting performed using the indicated antibodies (left panel). Molecular weight markers are indicated in kDa. Enrichment of Ku80 in chromatin fractions was quantified (lower panel; *n* = 3; individual data points are shown and error bars represent the SEM). **e** Restriction-Enzyme Mediated Integration REMI of plasmid DNA was evaluated in Ax2, *h3b*^*−*^ cells, or *h3b*^*−*^ cells expressing Flag-H3b^WT^ or Flag-H3b^S10T S28A^ (*n* = 3; individual data points are shown and error bars represent the SEM). Source data are provided in the Source Data file.

Having established that S10 and S28 of H3b are ADP-ribosylated in response to DSBs, we next wished to assess whether these modifications contribute to the ability of cells to repair DNA damage. Initially, we investigated the requirement for H3b ADPr in tolerance to DNA DSBs. Consistent with S10T or S28A mutations only partially reducing H3b ADPr, whilst expression of Flag-H3b^wt^ is able to complement the radiosensitivity of the *h3b*^*−*^ strain, expression of Flag-H3b^S10T^ and Flag-H3b^S28A^ only partially rescue this phenotype (Supplementary Fig. 4B). However, *h3b*^*−*^ cells expressing Flag-H3b^S10TS28A^ remain as sensitive to phleomycin as control cells, indicating that defective H3b ADPr at S10 and S28 is associated with a significant increase in sensitivity to DNA DSBs (Fig. 4a and Supplementary Fig. 4B). Additionally, whilst PARPi sensitise parental *h3b*^*−*^ cells expressing Flag-H3b^WT^ to phleomycin, they do not induce further radiosensitivity in Flag-H3b^S10TS28A^ expressing cells (Figs. 4b,  S5), indicating PARPs and H3S10/S28 likely function in the same pathway with regards radioresistance.

To assess whether this sensitivity to phleomycin correlates with defective DSB repair, the induction and decay of γH2AX were assessed in *h3b*^*−*^ cells complemented with either Flag-H3b^WT^ or Flag-H3b^S10T S28A^. Whilst *h3b*^*−*^ cells expressing Flag-H3b^S10T S28A^ exhibit a similar decay rate of γH2AX to those expressing Flag-H3b^WT^ following exposure to phleomycin it is delayed (Fig. 4c), indicating a requirement for ADPr at H3bS10 and S28 to initiate repair of DNA damage. To gain a mechanistic insight into this phenotype, we additionally assessed whether similar to Adprt1a-dependent ADPr^35^, H3bS10 and S28 ADPr are required for the PBZ domain-dependent enrichment of Ku at DNA lesions in response to DNA DSBs. As described previously, whilst Ku80 is enriched in chromatin prepared from Ax2 cells exposed to phleomycin, it is reduced in the *h3b*^*−*^ strain (Fig. 4d). In contrast to Flag-H3b^WT^, the expression of Flag-H3b^S10TS28A^ is unable to fully rescue this phenotype. Importantly, restriction-enzyme-mediated integration (REMI) of plasmid DNA into the genome (Fig. 4e), an event that is dependent on NHEJ^35,37^, is reduced in *h3b*^*−*^ cells. Moreover, whilst expression of Flag-H3b^WT^ can partially rescue this phenotype, Flag-H3b^S10TS28A^ is unable to do so. Together, these data demonstrate the direct role of H3b ADPr at S10 and S28 in DNA repair and in particular, the initiation of the NHEJ pathway through promoting the recruitment and/or stabilisation of the heterodimer Ku70/Ku80 at break sites.

### Histone ADP-ribosylation and control of mitotic progression

**Fig. 5:**
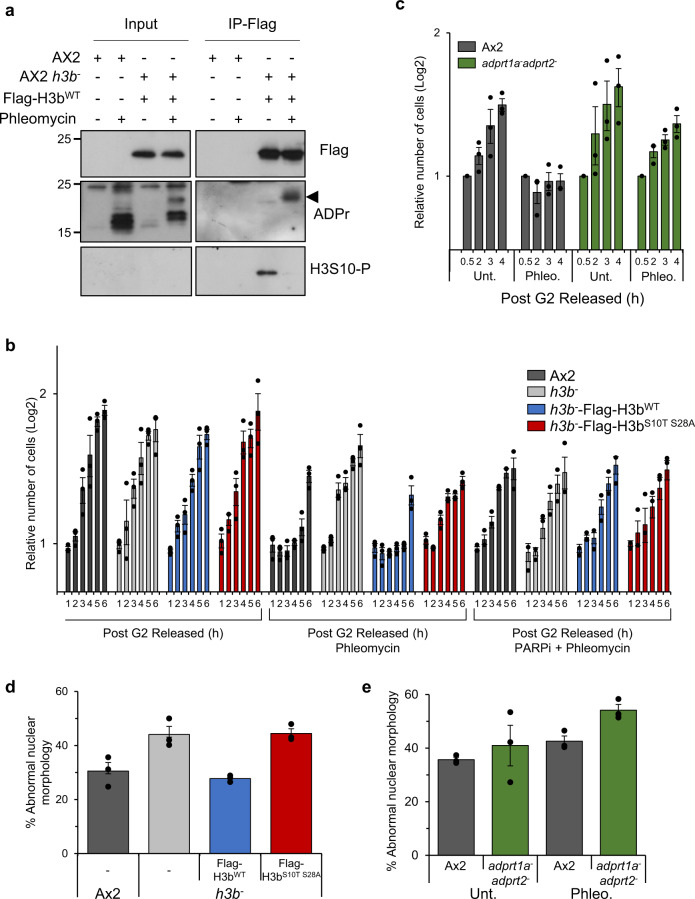
H3b ADP-ribosylation is required to suppress premature mitotic entry with DNA damage. **a** Ax2 or *h3b*^*−*^ cells expressing Flag-H3b^WT^ or Flag-H3b^S10TS28A^ were left untreated or exposed to phleomycin as indicated. Western blotting of Flag-immunoprecipitates was performed using the indicated antibodies. Molecular weight markers are indicated in kDa. **b** Ax2, *h3b*^*−*^ cells, or *h3b*^*−*^ cells expressing Flag-H3b^WT^ or Flag-H3b^S10T S28A^ were arrested in G2 and following treatment or not with phleomycin in the absence or presence of olaparib, released into fresh media to allow resumption of the cell cycle. Progression through mitosis was monitored by assessing cell number at times following release from G2. **c** Ax2 and *adprt1a*^*−*^*adprt2*^*−*^ cells were arrested in G2 and following treatment or not with phleomycin assessed for mitotic entry as described in (**b**). **d** Ax2, *h3b*^*−*^ cells and *h3b*^*−*^ cells expressing Flag-H3b^WT^ or Flag-H3b^S10TS28A^ were treated with a transient exposure to phleomycin for one h and after 48 h of recovery abnormal nuclear morphology assessed by microscopy. **e** Chromosome fragmentation in Ax2 or *adprt1a*^*−*^*adprt2*^*−*^ cells was assessed as in (**d**). All graphs represent the average of 3 independent experiments. Individual data points are shown and error bars represent the SEM. Source data are provided in the Source Data file.

Having established a role for H3b S10/S28 ADPr in DNA repair, we next wished to assess the relationship between this PTM with phosphorylation at these sites. Given that Ser-ADPr is able to disrupt phosphorylation at H3S10 in vitro^32^, we assessed whether these modifications exhibit opposing profiles in response to DNA damage. Consistent with previous observations, Flag-H3b^wt^ is ADP-ribosylated in response to DNA DSBs (Fig. 5a). Importantly, however, this is accompanied by a concomitant reduction in the levels of H3b phosphorylation at S10, indicating a reciprocal relationship between the levels of H3bS10 phosphorylation and ADPr in response to DNA damage.

Phosphorylation of histone H3S10 and S28 by the Aurora A and B kinases during mitosis has been implicated in chromatin condensation and mitotic progression in a number of organisms^53^. Given the reciprocal relationship between the levels of ADPr and phosphorylation at these sites, in addition to the availability of H3b S10 mutants that are ADPr-defective but phospho-competent, we assessed the consequences of defective ADPr on mitotic entry following DNA damage. To achieve this, cells were arrested in G2 and left either untreated, or exposed to phleomycin. Following removal of phleomycin and release from the G2 block, mitotic progression was assessed by monitoring synchronous cell doubling times. Consistent with the activation of a G2 checkpoint, a delay in mitotic entry is observed following exposure of Ax2 cells to phleomycin (Supplementary Fig. 6A). However, a more rapid resumption of cell cycle progression is apparent in the *h3b*^*−*^ strain relative to parental Ax2, indicating these cells enter mitosis prematurely following exposure to DNA DSBs (Fig. 5b and Supplementary Fig. 6B). Whilst expression of Flag-H3b^wt^ is able to restore the ability of cells to restrain mitosis in response to DNA damage, Flag-H3b^S10TS28A^ expression is unable to do so and these cells progress through mitosis significantly quicker following a transient induction of DNA damage (Fig. 5b) with persistent γH2AX (Supplementary Fig. 7). Moreover, whilst PARPi similarly induces premature mitotic entry following DNA damage in AX2 or *h3b*^*−*^ cells expressing Flag-H3b^wt^, they do not exacerbate this phenotype in *h3b*^*−*^ cells expressing H3b^S10TS28A^ (Fig. 5b and Supplementary Fig. 6C), indicating that PARP activity and H3S10/S28 ADPr function in the same pathway with regards restraining mitotic entry in response to DSBs. A prediction of this model is that cells defective in ADPr will similarly enter mitosis prematurely in response to DNA damage. Consistent with this, we observe that cells disrupted in the principal *Dictyostelium* DDR PARPs Adprt1a and Adprt2 (*adprt1a*^*−*^*adprt2*^*−*^ cells) similarly enter mitosis prematurely following a transient exposure to DNA DSBs (Fig. 5c). Taken together, these results suggest that ADPr of H3b at S10/S28 is required for an effective cell cycle checkpoint and that disruption of these events results in premature entry into mitosis with unresolved DNA damage.

Given the observed genome instability of *h3b*^*−*^ cells, we hypothesised that defective ADPr and mitotic entry with unresolved DSBs will contribute towards the increased genome instability observed in the *h3b*^*−*^ strain (Fig. 1a). To test this, we assessed nuclear morphology 48 h after a transient induction of DSBs in Ax2 cells or the *h3b*^*−*^ strain with or without expression of Flag-H3b^wt^ or Flag-H3b^S10T S28A^. Consistent with previous observations, we observe abnormal nuclear morphology in the *h3b*^*−*^ strain relative to AX2. Strikingly, whilst expression of Flag-H3b^wt^ is able to rescue this phenotype, Flag-H3b^S10T S28A^ is unable to do so (Fig. 5d). Given the premature mitotic entry of *adprt1a*^*−*^*adprt2*^*−*^ cells following DNA damage, we also assessed this phenotype in these cells. These cells similarly displayed abnormal nuclear morphology in response to DNA damage (Fig. 5e), further supporting a requirement for H3b ADPr in genome integrity.

**Fig. 6:**
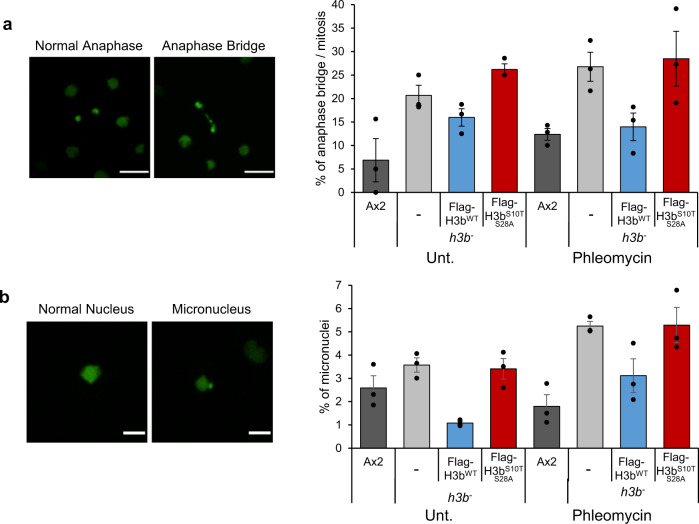
H3b ADP-ribosylation is required for faithful completion of cytokinesis. **a** Ax2, *h3b*^*−*^ cells, or *h3b*^*−*^ cells expressing Flag-H3b^WT^ or Flag-H3b^S10T S28A^ expressing GFP-H2B were assessed for anaphase bridges during cytokinesis in either untreated cells, or cells exposed to phleomycin. A representative image of an anaphase bridge is illustrated (left panel) and quantification of data from three independent experiments (right panel). Scale bars: 10 μm. **b** Ax2, *h3b*^*−*^ cells, or *h3b*^*−*^ cells expressing Flag-H3b^WT^ or Flag-H3b^S10T S28A^ expressing GFP-H2B were assessed for the presence of micronuclei in either untreated cells, or cells exposed to phleomycin. A representative image of a micronucleus is illustrated (left panel) and quantification of data from 3 independent experiments (right panel). Scale bars: 5 μm. Source data are provided in the Source Data file.

To further asses the consequences of deregulated H3b ADPr on mitotic progression, we performed live cell imaging in the *h3b*^*−*^ strain expressing either Flag-H3b^wt^ or Flag-H3b^S10T S28A^ to assess the ability of cells to undergo cytokinesis. The *h3b*^*−*^ strain exhibits difficulties completing cytokinesis either in untreated cells, or cells exposed to phleomycin, a phenotype that is reflected in these cells displaying elevated levels of anaphase bridges (Fig. 6a and Supplementary Movie S1). This difference is apparent in untreated cells, suggesting some difficulties in completing cytokinesis independently of exogenous DNA damage. Nevertheless, whilst the levels of DNA damage-induced anaphase bridges are reduced by expression of Flag-H3b^wt^, this is not apparent in *h3b*^*−*^ cells expressing Flag-H3b^S10T S28A^. We also observed elevated levels of micronuclei in the *h3b*^*−*^ strain relative to Ax2 (Fig. 6b and Supplementary Movie S2). Whilst these phenotypes are partially complemented by the expression of Flag-H3b^wt^, no such reduction in the number of micronuclei is observed on the expression of Flag-H3b^S10T S28A^, indicating a requirement for H3b ADPr in suppressing micronuclei formation. Overall, these data indicate ADPr of H3b is required to regulate DNA repair and restrain mitotic entry in response to DNA damage. Furthermore, they also implicate H3b ADPr in the faithful completion of cytokinesis to maintain genome instability.

## Discussion

The role of PARPs in regulating DNA repair is well established. However, despite DNA damage-induced ADPr being described decades ago, the mechanistic basis of how site-specific ADPr events regulate DNA repair and genome stability remains elusive. This, in part, has been due to the lack of appropriate technologies to identify ADPr events induced in response to different types of DNA damage. Whilst recent developments in MS approaches have resolved this issue^21–25^, understanding the functional significance of these modifications remains a challenge, particularly in the context of histones. The identification that serine is the major target for ADPr in response to genotoxic stress and that this modification can influence other PTMs on histone tails introduces an added level of complexity to the system. This is compounded by difficulties in manipulating multi-copy histone genes in vertebrates, or the limited conservation of PARPs in model organisms where this is possible. *Dictyostelium* is an ideal experimental platform to bridge this gap due to the high degree of vertebrate DNA repair pathway conservation in this organism, including PARPs, and the ability to manipulate single copy histone genes in this system. Here we exploit these characteristics to assess the role of histone ADPr in DNA repair. Importantly, we develop a separation of function mutation strategy that allows us to assess the role of Ser-ADPr in DNA repair and cell cycle progression whilst maintaining the phosphorylation status of these sites.

In vertebrates, ADPr was originally believed to be confined to glutamate and aspartate, and we have similarly been able to identify ADPr at these sites in *Dictyostelium*, most notably at H2BE18/19^50^. However, ADPr of histones at serine has emerged as a major event in response to genotoxic stress^23,31^. The recent identification that this is predominantly mono-ADPr catalysed by PARP1/HPF1 that can be reversed by ADP-ribosylhydrolase 3 (ARH3) has more fully defined the readers and writers of the histone ADPr code^24,30,54^. Using an antibody that recognises HPF1-dependent serine-ADPr^34^ coupled with mutation strategies, we identify that *Dictyostelium* proteins, including histones, are also ADP-ribosylated on serine (Figs. 2, 3). These observations indicate Ser-ADPr is conserved in *Dictyostelium* suggesting it is widely conserved in eukaryotes. HPF1 is critical in converting the catalytic activity of PARP1 and PARP2 from Asp/Glu to serine^24,30^. Intriguingly, however, we have been unable to identify an HPF1 homologue in the *Dictyostelium* genome. This might indicate the presence of an unidentified factor in *Dictyostelium* that acts analogous to HPF1 to convert the catalytic activity of the principal DDR PARPs (Adprt1a/Adprt2) to modify serine. Alternatively, the findings that HPF1 and PARP1 act together to form a full active site for the enzyme^55,56^, taken together with our observations that Adprt1a catalyses mono-ADPr in vitro^50^, raise the possibility that *Dictyostelium* PARPs might contain an intrinsic Ser-ADPr activity, circumventing the need for HPF1. Distinguishing these possibilities will provide insights into the nature of PARP catalytic specificity in a number of organisms.

The identification of Ser-ADPr introduces a further level of regulation through influencing PTMs at adjacent amino acids, or at serine directly. This is a particularly attractive hypothesis in the context of N-terminal histone tails where the high abundance of multiple PTM sites will allow ADPr to impact on a wide variety of modifications. Whilst this concept was originally proposed in the context of Ser-ADPr^32,33^, it has recently been extended to glutamate ADPr^26^. Therefore, different patterns of ADPr may impact on a wide variety of PTMs, introducing additional flexibility in the histone code. The mutually exclusive modification of serine by ADPr or phosphorylation^32,34^ taken together with the widespread use of serine phosphorylation to regulate a large number of pathways, makes it likely the interplay between these opposing PTMs will impact on a variety of processes. However, the analysis of the relationships is challenging. Commonly used mutation strategies that disrupt ADPr will also interfere with serine phosphorylation making it difficult to disentangle the functional significance of these opposing PTMs. Phosphorylation events may also be critical for cell viability, a possibility that is highlighted by a large number of DNA damage-induced Ser-ADPr sites also being targets for the mitotic kinases Aurora A and B^25,52^. Whilst generating phospho-mimic mutations may address these problems, these strategies employ acidic amino acids (e.g. Asp) that are also ADP-ribosylated. By exploiting the inability of threonine to be ADPr we have developed a mutation strategy that allows the disruption of site-specific ADPr events whilst retaining the ability to maintain phosphorylation at these sites. Although the ability to phosphorylate S-T sites may be context dependent, we predict this strategy will be applicable to other ADPr sites in histones and other targets for Ser-ADPr. The conservation of the major histone PTMs in *Dictyostelium*^45–49^, together with the ability to generate site-specific mutations in histones, makes this an attractive system to study these possibilities.

Our ability to manipulate histone genes in *Dictyostelium* has provided unique insights into the relationship between histone ADPr and DNA repair. The deletion of *h3b* is not lethal to *Dictyostelium* cells. Strikingly, however, these cells display chromosomal instability, a phenotype that is reflected in radiosensitivity and a delay in resolving DNA DSBs. Our data clearly indicate a requirement for S10/S28 ADPr in supressing these phenotypes (Fig. 4), as opposed to a more general requirement for H3b in these processes. Mechanistically, our data point to a link between H3b ADPr and accumulation of Ku at DNA lesions. It is well established that accumulation of DNA repair and chromatin remodelling factors is achieved in a PARP-dependent manner though ADP-ribose binding motifs in these proteins^1^. Our previous work identified this mechanism is conserved in *Dictyostelium* with the PBZ domain in Ku70 being required to assemble DNA repair factors at damage sites to facilitate NHEJ^35^. However, the substrates modified at sites of DNA damage that enable the accumulation of repair factors at DNA damage are unknown. Here we resolve this question by illustrating that ADPr of H3b at S10/S28 is required for the accumulation of Ku at DSBs. Intriguingly, PBZ domains interact with poly-ADPr, whilst Ser-ADPr is predominantly mono-ADPr. Whether the requirement for both a PBZ domain and H3b Ser-ADPr to assemble Ku at breaks represents poly-ADPr of H3bS10/28 or other sites/factors in *Dictyostelium* is unknown. Additionally, it is possible that this indicates a direct link between H3b ADPr and recruitment of repair factors such as Ku to DNA lesions. However, a wide variety of histone PTMs, including methylation, acetylation and ubiquitylation are required for efficient resolution of DSBs. Therefore, another non-mutually exclusive possibility is that S10/S28 ADPr impacts on other histone PTMs required for DSB repair either directly, or by influencing the accumulation of histone modifying activities at DSBs. The conservation of key human repair and chromatin modifications in *Dictyostelium*, along with the ability to manipulate single copy histone genes in this experimental system, suggests this organism will be an important model to investigate these possibilities.

Our mutation strategy that retains phosphorylation at H3S10 while disrupting ADPr reveals a critical role for ADPr of H3bS10/S28 to restrain mitotic entry in response to DNA damage. It is possible that similar to recruiting repair factors to DSBs, S10/S28 ADPr promotes the accumulation and activation of checkpoint proteins to damage sites. In this regard, the PAR-binding PBZ-module of CHFR is required for a functional antephase checkpoint that restrains mitotic entry in response to a variety of stresses^57^. However, it is also possible ADPr has a wider impact on cell cycle progression in response to DNA damage. Phosphorylation of S10 and S28 by Aurora kinases is pivotal in regulating various aspects of mitosis including chromosome condensation^58–60^ and initiating a phospho-methyl switch that causes displacement of HP1 and PRC1 from chromatin^61,62^. Our data indicate a reciprocal relationship in the levels of H3bS10 ADPr and phosphorylation following DNA damage. This may reflect DNA damage-induced ADPr and loss of the mitotic cell population by activation of the G2/M checkpoint. However, given Ser-ADPr can directly inhibit ADPr^32^, it is also possible that ADPr counteracts S10/S28 phosphorylation to inhibit normal cell cycle progression and mitosis. ADPr of S10/S28 may also interfere with other PTMs at adjacent sites that are required for cell cycle progression, such as T3 phosphorylation that promotes the enrichment of CPC at centromeres that in turn phosphorylates H3S10/S28 to promote mitotic progression^63,64^. Indeed, many of the sites ADP-ribosylated in response to DNA damage are targets for mitotic kinases and Aurora B activity is inhibited in response to DNA damage through ADPr^65^. This might suggest a wider role of ADPr in regulating mitotic progression through a variety of mechanisms. In support of this model, we observe that H3b-ADPr mutants or *adprt1a*^*−*^*adprt2*^*−*^ cells exhibit difficulties undergoing cytokinesis, even in the absence of exogenous DNA damage (Figs. 5 and 6). However, these phenotypes could also be explained by an accumulation of DNA damage in DNA repair defective strains. For example, it has been known for some time that PARPs and ADPr function in regulating mitosis^66^. Cells defective in the PARP1 and/or PARP2 exhibit a variety of mitotic defects including centrosome amplification, ultra-fine anaphase bridges and loss of spindle assembly checkpoint integrity^66–71^. However, these phenotypes can often be caused by defective replication-associated repair, a mechanism that both PARP1 and PARP2 regulate^5,16–19^. As such, defective NHEJ in H3b-ADPr mutants may also result accumulation of endogenous DNA damage that subsequently results in difficulties during cytokinesis. Therefore, we are currently unable to distinguish whether the genome instability in H3b-ADPr mutants is a consequence of defective cytokinesis, DNA repair, or both. However, our findings that H3b^S10TS28A^ mutants or *adprt1a/adprt2* cells display premature entry into mitosis in response to DNA damage provide a clear link between DNA damage responsive PARPs, histone ADPr and regulation of mitotic entry in response to DNA damage.

In summary, we observe that the histone variant H3b is required for efficient DNA repair and genome stability in *Dictyostelium*. Through identifying that Ser-ADPr is conserved in this organism, we identify that the requirement for H3b in DSB repair is through ADPr at S10 and S28 and define the mechanistic basis of this regulation by illustrating it promotes the PARP-dependent assembly of NHEJ factors at DSBs. Critically, we also identify that ADPr of H3S10/S28 is required to restrain mitotic entry in the presence of DNA damage indicating a critical role for ADPr in regulating cell cycle progression in response to DNA damage.

## Methods

### Cell culture, DNA transfection, establishment of stable cell lines

*Dictyostelium* Ax2 cells were grown according to standard procedures, either axenically with HL5 media (Formedium^®^ HLB0101; 20% Glucose) or on SM agar plates in association with *Klebsiella aerogenes* at 22 °C. Exponential growing cells are defined as a concentration of 2–6 × 10^6^ cells/ml.

The *h3b*^*−*^, *adprt1a*^*−*^*adprt2*^*−*^, *ku80*^*−*^ and e*xo1*^*−*^ strains have been previously described^35,37,38,42,45^. The Flag-Rad51 expression plasmid has been previously described^42^.

H3b cDNA was cloned into the plasmid pDM304-Cter 3x-Flag using the following primers (forward: GCAGATCTAAAATGGCCCGTACAAAACAAACCG; reverse: GCACTAGTAGCACGTTCACCTCTGATACGTCTGG).

Mutagenesis was performed using *QuikChange XL Site-Directed Mutagenesis Kit*^®^ and appropriate primers as followed: *H3b S28A (forward:* CTTCTCAAAAAGCTTTTCCATCAACCCAAGG; reverse: CCTTGGGTTGATGGAAAAGCTTTTTGAGAAG), H3b S10A (forward: CAAACCGCTAGAAAAGCAACTGGTGCTAAAGTACC; reverse: GGTACTTTAGCACCAGTTGCTTTTCTAGCGGTTTG), H3b S10T (forward: CAAACCGCTAGAAAAACAACTGGTGCTAAAGTACC; reverse GGTACTTTAGCACCAGTTGTTTTTCTAGCGGTTTG).

Transfection was performed as previously described^43^, using the *GenePulser Xcell*^®^ (*Biorad*^®^). Transfected cell lines were selected with the antibiotic G418 (10 µg/ml) for at least 7 days to establish stable cell lines. Stable cell lines were cultivated with media complemented with G418 antibiotic (10 µg/ml).

### Acid extraction

Acid extraction was performed as previously described^50^. Briefly, exponential growing cells were collected and resuspended at a concentration of 1 × 10^7^ cells/ml and either untreated or exposed to 1 h of phleomycin *(*300 µg/ml). Cells were pelleted, washed twice with cold KK2, and resuspend in lysis buffer (50 mM Tris HCl pH 8.0, 10 mM NaCl, 3 mM MgCl_2_, 3 mM CaCl_2_, 0.5 M Sorbitol, 0.6% Triton X-100; completed with proteases (Complete cocktail-Roche^®^), phosphatases (Na_3_VO_4_ and NaF), PARP (50 μM Benzamide) and PARG (200 μM DEA) inhibitors) at a concentration of 1 × 10^8^ cells/ml, incubated with rotation at 4 °C for 10 min, and the nuclei pelleted by centrifugation at 2300 *g* for 5 min. Extracted nuclei were resuspended/washed in nuclear extraction buffer containing 4 M urea, and 2% β-mercaptoethanol, and pelleted again by centrifuging at 2300 *g* for 5 min. Isolated nuclei were then resuspended in 0.4 N HCl at a density of 2 × 10^8^ nuclei/ml, and mixed by rotation at 4 °C for 1 h. Acid extracted histones were harvested by centrifugation at 16,000 *g*, and the supernatant precipitated by addition of 6.5 volumes of cold acetone, incubating at −20 °C over-night, and centrifuging at 16,000 *g* for 15 min at 4 °C. The pellet was washed twice with cold acetone, air-dried, and resuspended first in water at 37 °C for 15 min, then SDS buffer (10 mM Tris pH 6.8, 5% glycerol, 1% SDS, 1% β-Mercaptoethanol) was added vol/vol and boiled at 95 °C for 5 min.

### Biochemical fractionation

Cells were treated and collected for acid extraction. Cell pellets were lysed 5 min at 4 °C with buffer A (10 mM Tris HCl pH 8.0, 10 mM KCl, 1.5 mM MgCl_2_, 3 mM CaCl_2_, 0.34 M sucrose, 10% glycerol, 0.5% Triton X-100; completed with proteases (Complete cocktail-Roche^®^), phosphatases (Na3VO4 and NaF), PARP (50 μM Benzamide) and PARG (200 μM DEA) inhibitors) at a concentration of 1 × 10^8^ cells/ml. Cell lysis lysates were centrifuged for 5 min at 1700 *g*, 4 °C. Supernatants (cytoplasm) were collected, and the nuclear pellet was washed once with lysis buffer A (including inhibitors). The nuclear pellets were resuspended in no-salt buffer (3 mM EDTA, 0.2 mM EGTA, inhibitors as in lysis buffer) and hypotonic lysis left for 30 min at 4 °C, with occasional vortexing. Samples were centrifuged for 5 min at 8000 *g*, 4 °C, the supernatants were removed, and the chromatin pellets were washed once with no-salt buffer. The final pellets were resuspended in SDS buffer (10mMTris 1 M pH 6.8, 5% glycerol, 1% SDS, 1% β-Mercaptoethanol at a concentration of 1 × 10^8^ cells/ml, and boiled at 95 °C for 5 min and were called the chromatin fraction.

### Immunoprecipitation

For asynchronous cells, immunoprecipitations were performed on chromatin fractions prepared as described above, while for mitotic cells, they were performed on whole-cell extract. Chromatin pellets or cell pellets were resuspended in 4% SDS buffer (4% SDS, 100 mM Tris HCl pH6.8) and boiled at 95 °C for 10 min to denature the proteins. An aliquot was taken as the input. The remaining samples were diluted 10 times in IP buffer (50 mM Tris HCl pH8, 200 mM NaCl, 1 mM EDTA, 1 mM DTT, 10% glycerol, 1% Triton X-100 + inhibitors). 50 µl of anti-Flag-M2 affinity gel (Sigma^®^, A2220) were used per immunoprecipitation. Prior adding sample onto the beads, they were washed twice with IP buffer. Diluted samples were incubated with the beads for 2 h at 4 °C on a rotating wheel. After centrifugation (500 *g*, 2 min), the beads were washed three times with IP buffer and once with TBS buffer (50 mM Tris-HCl pH 7.4, 150 mM NaCl). Elution through competition using Flag peptide (Sigma^®^, F4799) was performed: an equal volume of 100 ng/µl Flag-peptide in TBS buffer was added to the beads, the samples were vortexed gently for 5 min and centrifuged to collect the supernatant. This was repeated a second time. The eluates were diluted in SDS buffer and boiled before immunoblot analysis.

### Affinity purification of ADPr proteins

Cells were pelleted, washed with cold KK2, and resuspended in lysis buffer (50 mM Tris HCl pH 8.0, 10 mM NaCl, 3 mM MgCl_2_, 3 mM CaCl_2_, 0.5 M Sorbitol, 0.6% Triton X-100; with protease inhibitors (Complete cocktail-Roche^®^), PARP (50 μM Benzamide) and PARG (200 μM DEA) inhibitors) at a concentration of 100 × 10^6^ cells/ml, incubated with rotation at 4 °C for 10 min, and the nuclei pelleted by centrifugation at 2300 *g* for 5 min. Extracted nuclei were resuspended/washed with the same buffer. 10% of the nuclei were collected as the Input. The rest of the nuclei were lysed with GnHCl buffer (6 M Guanidine hydrochloride, 50 mM TrisPh8.5) for 30 min with rotation at 4 °C. Then, the samples were diluted 10 times in IP buffer (50 mM Tris HCl pH8, 200 mM NaCl, 1 mM EDTA, 1 mM DTT, 10% glycerol, 1% Triton X-100 + inhibitors). 20 µl of prior washed Dynabead^®^-Protein G (Invitrogen^®^) was added for 1 h. Meanwhile, 3 µg of PAN-ADPr reagent (Merk^®^ MABE1016) was incubated with 30 µl of Dynabead^®^-Protein G (Invitrogen^®^) for 1 h in 0.01% Tween-20 in KK2. Pre-cleared samples were incubated with the conjugated beads at 4 °C on a rotating wheel. The beads were washed for 5 min, with the following buffers: W1 (Tris pH8 10 mM, KCl 150 mM, NP40 0.5%, EDTA 1 mM), W2 (Tris pH8 10 mM, NaCl 200 mM, TritonX100 0.5%) W3 (Tris pH8 10 mM, NaCl 400 mM, TritonX100 0.5%), W4 (Tris pH8 10 mM, NaCl 500 mM, TritonX100 0.5%), W5 (Tris pH8 10 mM, LiCl 250 mM, NP40 0.5%, EDTA 1 mM) and W6 (Tris pH8 10 mM, EDTA 1 mM). Elution was performed by boiling samples in SDS buffer. Samples were analysed by immunoblotting.

### Cell synchronisation

Cells were arrested in G2 phase as previously described^40^. Briefly, exponentially growing cells were seeded at a dilution of 1 × 10^6^cells/ml and incubated in shaking suspension (220 rpm) at 9.5 °C over-night. Subsequently, cells were released from this block by raising the temperature of cultures to 22 °C within 30 s and incubating cells in shaking suspension at 22 °C.

To enrich cell population in mitosis, cells were first arrested in G2, then released in presence of 10 µg/ml of nocodazole (Cayman Chemical Company^®^). For biochemical study, cells were collected 4 to 6 h after the release.

To assess entry in mitosis following DNA damage, cells were arrested in G2 as above and treated with 200 µg/ml of phleomycin for 1 h, then washed twice with 9.5 °C KK2 (centrifugation at 10 °C) and released from cell cycle arrest by adding 22 °C HL5 media.

To monitor cell proliferation after G2 release, cells were plated on a 8-well slide (Starsted^®^) at 22 °C and recorded for approximately 10 h with a microscope Zeiss^®^ IX71 (10X objective, brightfield, 1 picture/min). Using the software ImageJ^®^, cell number was manually counted on pictures of the appropriate time points and normalised with the earlier time point.

### Sensitivity assays

Sensitivity assays were performed as described previously^35^. Exponentially growing *Dictyostelium* were diluted at a concentration of 1 × 10^6^ cells/ml in HL5 media and treated for one h with phleomycin at the indicated concentrations. Afterwards cells were diluted in KK2, 250 cells were mixed with *K. aerogenes*, and spread onto 140 mm SM agar plates. The plates were incubated at 22 °C. After 4 to 7 days, *Dictyostelium* plaque formation were counted and cell survival calculated by normalising with the number of colonies without phleomycin treatment. Graphs of data were constructed using Excel 2016.

### PARP inhibitors

Two PARPs inhibitors were used in this study Olaparib (LKT laboratories^®^; stock solution 10 mg/ml in DMSO, experimental concentration 230 µM) and Benzamide (Sigma^®^; freshly prepared stock of 500 mM in 70% ethanol, experimental concentration 2.5 mM). PARP inhibitors were added one-hour prior to phleomycin treatment and during application of DNA damage.

### Restriction-enzyme-mediated integration of plasmid DNA into the *Dictyostelium* genome

REMI was performed essentially as previously described^37^. To generate vectors bearing restriction-enzyme sites at DNA ends, the plasmid pHygTm(plus)/pG7 (containing a Hygromycin cassette) was cut with BamH1 (NEB), treated with calf intestinal alkaline phosphatase (NEB) and purified on PCR clean up columns (QIAGEN^®^). 4.5 × 10^6^ cells per condition were transfected with 2 µg of *BamH*1-linearised construct with or without 20 units of the restriction-enzyme BamH1 (NEB). Electroporated cells were spread on four 140 mm plates with axenic HL5 media. 24 h after transfection, hygromycin (35 μg/ml) was added for 5 days. Afterwards, plates were washed with KK2, fixed with 3.7% formaldehyde and stained with Crystal Violet solution. Colonies were counted and REMI induction calculated as the ratio of colony numbers with and without *BamH*1. Graphs of data were constructed using Excel 2016.

### Immunoblot analysis

Proteins were resolved by Mini Gel SDS-PAGE (Biorad^®^ system) and transferred to PVDF membrane (Immobilon-P^®^- Millipore^®^) according to standard procedures. Blocking and antibody incubations were performed in TBS - 0.2% Tween-20^®^ 5% milk (Marvel^®^). The following primary antibodies were used: anti-Ku80, (1:1000)^38^, anti-actin (1:5000, Santa-Cruz^®^ sc-1615), anti-H2AX-P (1:1000, Abcam® ab11174), anti-H3 (1:2000, Abcam^®^ ab12079), anti-flag (1:2000, SIGMA^®^ F1804 and F2425), anti-mono-ADPr AbD33205 (1:1000)^34^, Anti-H3S10-p (1:1000, Bethyl^®^ A301-844A-T, and Abcam^®^ ab5176). Global ADP-ribosylation sigma was detected using the reagent anti-PAN-ADPr (1:2000, Merk^®^ MABE1016). Appropriate HRP-conjugated secondary antibodies were used anti-mouse (1;10000, DAKO^®^), anti-rabbit (1;10000, DAKO^®^), anti-goat (1;10000, DAKO^®^) and anti-human (1:5000)^34^. Immuno-reactive bands were detected by chemo-luminescence induced by Immobilon^®^ western substrate (Millipore^®^), detected with the LI-COR^®^ Odyssey-Fc machine and quantified using Image-Studio^®^ software.

### Immunofluorescence microscopy

Cells were plated onto glass coverslips and allowed to attach. Cells were fixed with 4% paraformaldehyde (AlfaAesar^®^) for 15 min. Where necessary, prior to fixation, soluble protein was pre-extracted using 0.5% Triton X-100 in KK2 for 5 min at 4 °C. Cells were then permeabilised in 0.5% Triton X-100 in KK2 for 10 min, and blocked in 1% bovine serum albumin (Sigma^®^) for 1 h. Coverslips were stained with primary antibody (2 h, room temperature) against H3S10-P (1:500, Bethyl^®^ A301-844A-T) or γH2AX (1/500, Abcam^®^ ab11174), washed extensively in KK2-0.01% Tween-20^®^, and stained with fluorescently labelled secondary antibody (2 h, room temperature) anti-mouse FITC (1:500, DAKO^®^, F0232) or anti-rabbit TRITC (1:500, DAKO^®^, R0156). Following further washing, cells were mounted onto slides in Vectashield^®^ containing DAPI (Vector Laboratories). Samples were visualised using a microscope Zeiss IX71 equipped with a 10X dry objective and a 100X oil immersion objective lens and a Hamamatsu^®^ Orca-R^2^ camera. Pictures were analysed with ImageJ^®^ software.

### Live cell imaging

To monitor mitosis, we expressed GFP-H2B in the cells. Asynchronous cells were treated with 100 μg/ml of phleomycin for 1 h, then washed twice with LoFlo (Formedium^®^ LF1001). The cells were transferred on LoFlo agar, then squares of the agar were excised and inverted onto glass bottom imaging dishes and covered with mineral oil. Cells were imaged on a Nikon A1R confocal microscope with a ×60 1.4 NA oil objective. 3D stacks were captured at multiple positions every 30 s between 2 and 6 h after phleomycin treatment. Images were analysed as 2D projections of the original 3D stacks with Volocity software, version 6.3 (PerkinElmer). Graphs of data were constructed using Excel 2016.

### Reporting summary

Further information on research design is available in the Nature Research Reporting Summary linked to this article.

## Supplementary information

Supplementary Information

Description of additional Supplementary File

Supplementary Movie 1

Supplementary Movie 2

Reporting Summary

## Data Availability

The datasets generated during and/or analysed during the current study are available from the corresponding author on reasonable request. Source data are provided as a Source Data file. Source data are provided with this paper.

## Source data

Source Data

## References

[1] Gibson BA, Kraus WL (2012). New insights into the molecular and cellular functions of poly(ADP-ribose) and PARPs. Nat. Rev. Mol. Cell Biol..

[2] Martin-Hernandez K, Rodriguez-Vargas JM, Schreiber V, Dantzer F (2017). Expanding functions of ADP-ribosylation in the maintenance of genome integrity. Semin. Cell Dev. Biol..

[3] Azarm K, Smith S (2020). Nuclear PARPs and genome integrity. Genes Dev..

[4] Caldecott KW (2008). Single-strand break repair and genetic disease. Nat. Rev. Genet..

[5] Ronson GE (2018). PARP1 and PARP2 stabilise replication forks at base excision repair intermediates through Fbh1-dependent Rad51 regulation. Nat. Commun..

[6] Hanzlikova H, Gittens W, Krejcikova K, Zeng Z, Caldecott KW (2017). Overlapping roles for PARP1 and PARP2 in the recruitment of endogenous XRCC1 and PNKP into oxidized chromatin. Nucleic Acids Res..

[7] Hewitt, G. et al. Defective ALC1 nucleosome remodeling confers PARPi sensitization and synthetic lethality with HRD. *Mol. Cell* **81**, 767–783 (2020). 10.1016/j.molcel.2020.12.006 PMC7895907 33333017

[8] Blessing C (2020). The oncogenic helicase ALC1 regulates PARP inhibitor potency by trapping PARP2 at DNA breaks. Mol. Cell.

[9] Juhasz, S. et al. The chromatin remodeler ALC1 underlies resistance to PARP inhibitor treatment. *Sci. Adv.* **6**, eabb8626 (2020). 10.1126/sciadv.abb8626 PMC11206534 33355125

[10] Verma, P. et al. ALC1 links chromatin accessibility to PARP inhibitor response in homologous recombination-deficient cells. *Nat. Cell Biol.* **23**, 160–171 (2021). 10.1038/s41556-020-00624-3 PMC7880902 33462394

[11] Rulten SL (2011). PARP-3 and APLF function together to accelerate nonhomologous end-joining. Mol. Cell.

[12] Beck C (2014). PARP3 affects the relative contribution of homologous recombination and nonhomologous end-joining pathways. Nucleic Acids Res..

[13] Luijsterburg MS (2016). PARP1 links CHD2-mediated chromatin expansion and H3.3 deposition to DNA Repair by non-homologous end-joining. Mol. Cell.

[14] McVey M, Lee SE (2008). MMEJ repair of double-strand breaks (director’s cut): deleted sequences and alternative endings. Trends Genet.: TIG.

[15] Hanzlikova H (2018). The importance of Poly(ADP-Ribose) polymerase as a sensor of unligated Okazaki fragments during DNA replication. Mol. Cell.

[16] Bryant HE (2009). PARP is activated at stalled forks to mediate Mre11-dependent replication restart and recombination. EMBO J..

[17] Sugimura K, Takebayashi S, Taguchi H, Takeda S, Okumura K (2008). PARP-1 ensures regulation of replication fork progression by homologous recombination on damaged DNA. J. Cell Biol..

[18] Yang YG, Cortes U, Patnaik S, Jasin M, Wang ZQ (2004). Ablation of PARP-1 does not interfere with the repair of DNA double-strand breaks, but compromises the reactivation of stalled replication forks. Oncogene.

[19] Berti M (2013). Human RECQ1 promotes restart of replication forks reversed by DNA topoisomerase I inhibition. Nat. Struct. Mol. Biol..

[20] Hottiger MO (2015). Nuclear ADP-ribosylation and its role in chromatin plasticity, cell differentiation, and epigenetics. Annu. Rev. Biochem..

[21] Jungmichel S (2013). Proteome-wide identification of poly(ADP-Ribosyl)ation targets in different genotoxic stress responses. Mol. Cell.

[22] Zhang Y, Wang J, Ding M, Yu Y (2013). Site-specific characterization of the Asp- and Glu-ADP-ribosylated proteome. Nat. Methods.

[23] Leidecker O (2016). Serine is a new target residue for endogenous ADP-ribosylation on histones. Nat. Chem. Biol..

[24] Bonfiglio JJ (2017). Serine ADP-ribosylation depends on HPF1. Mol. cell.

[25] Larsen SC, Hendriks IA, Lyon D, Jensen LJ, Nielsen ML (2018). Systems-wide analysis of serine ADP-ribosylation reveals widespread occurrence and site-specific overlap with phosphorylation. Cell Rep..

[26] Huang D (2020). Functional interplay between histone H2B ADP-ribosylation and phosphorylation controls adipogenesis. Mol. cell.

[27] Chen, Q. et al. ADP-ribosylation of histone variant H2AX promotes base excision repair. *EMBO J.* **40**, e104542 (2020). 10.15252/embj.2020104542 PMC7809701 33264433

[28] Karch KR, Langelier MF, Pascal JM, Garcia BA (2017). The nucleosomal surface is the main target of histone ADP-ribosylation in response to DNA damage. Mol. Biosyst..

[29] Gibson BA (2016). Chemical genetic discovery of PARP targets reveals a role for PARP-1 in transcription elongation. Science.

[30] Gibbs-Seymour I, Fontana P, Rack JG, Ahel I (2016). HPF1/C4orf27 is a PARP-1-interacting protein that regulates PARP-1 ADP-ribosylation activity. Mol. Cell.

[31] Palazzo L. et al. Serine is the major residue for ADP-ribosylation upon DNA damage. *Elife* **7**, e34334 (2018). 10.7554/eLife.34334 PMC5837557 29480802

[32] Bartlett E (2018). Interplay of histone marks with serine ADP-ribosylation. Cell Rep..

[33] Liszczak G, Diehl KL, Dann GP, Muir TW (2018). Acetylation blocks DNA damage–induced chromatin ADP-ribosylation. Nat. Chem. Biol..

[34] Bonfiglio JJ (2020). An HPF1/PARP1-based chemical biology strategy for exploring ADP-ribosylation. Cell.

[35] Couto CA (2011). PARP regulates nonhomologous end joining through retention of Ku at double-strand breaks. J. Cell Biol..

[36] Hsu DW, Gaudet P, Hudson JJ, Pears CJ, Lakin ND (2006). DNA damage signaling and repair in Dictyostelium discoideum. Cell Cycle.

[37] Hsu DW (2011). DNA double-strand break repair pathway choice in Dictyostelium. J. Cell Sci..

[38] Hudson JJ (2005). DNA-PKcs-dependent signaling of DNA damage in Dictyostelium discoideum. Curr. Biol..

[39] Pears CJ, Lakin ND (2014). Emerging models for DNA repair: Dictyostelium discoideum as a model for nonhomologous end-joining. DNA Repair (Amst.).

[40] Couto CA (2013). Nonhomologous end-joining promotes resistance to DNA damage in the absence of an ADP-ribosyltransferase that signals DNA single strand breaks. J. Cell Sci..

[41] Gunn AR (2016). The role of ADP-ribosylation in regulating DNA interstrand crosslink repair. J. Cell Sci..

[42] Kolb AL, Gunn AR, Lakin ND (2017). Redundancy between nucleases required for homologous recombination promotes PARP inhibitor resistance in the eukaryotic model organism Dictyostelium. Nucleic Acids Res..

[43] Kolb AL (2018). Dictyostelium as a model to assess site-specific ADP-ribosylation events. Methods Mol. Biol..

[44] Dubin M, Fuchs J, Graf R, Schubert I, Nellen W (2010). Dynamics of a novel centromeric histone variant CenH3 reveals the evolutionary ancestral timing of centromere biogenesis. Nucleic Acids Res..

[45] Hsu DW, Chubb JR, Muramoto T, Pears CJ, Mahadevan LC (2012). Dynamic acetylation of lysine-4-trimethylated histone H3 and H3 variant biology in a simple multicellular eukaryote. Nucleic Acids Res..

[46] Stevense M, Chubb JR, Muramoto T (2011). Nuclear organization and transcriptional dynamics in Dictyostelium. Dev. Growth Differ..

[47] Chubb JR (2006). Developmental timing in Dictyostelium is regulated by the Set1 histone methyltransferase. Dev. Biol..

[48] Sawarkar R, Visweswariah SS, Nellen W, Nanjundiah V (2009). Histone deacetylases regulate multicellular development in the social amoeba Dictyostelium discoideum. J. Mol. Biol..

[49] Lohia R (2018). Deletion of Dictyostelium discoideum Sir2A impairs cell proliferation and inhibits autophagy. J. Biosci..

[50] Rakhimova A (2017). Site-specific ADP-ribosylation of histone H2B in response to DNA double strand breaks. Sci. Rep..

[51] Asano, Y. et al. Knock-in and precise nucleotide substitution using near-PAMless engineered Cas9 variants in Dictyostelium discoideum. *Sci Rep* **11**, 11163 (2021). 10.1038/s41598-021-89546-0 PMC8159936 34045481

[52] Hendriks IA, Larsen SC, Nielsen ML (2019). An advanced strategy for comprehensive profiling of ADP-ribosylation sites using mass spectrometry-based proteomics. Mol. Cell Proteom..

[53] Wang F, Higgins JM (2013). Histone modifications and mitosis: countermarks, landmarks, and bookmarks. Trends Cell Biol..

[54] Fontana, P. et al. Serine ADP-ribosylation reversal by the hydrolase ARH3. *Elife* **6**, e28533 (2017). 10.7554/eLife.28533 PMC5552275 28650317

[55] Suskiewicz MJ (2020). HPF1 completes the PARP active site for DNA damage-induced ADP-ribosylation. Nature.

[56] Sun FH (2021). HPF1 remodels the active site of PARP1 to enable the serine ADP-ribosylation of histones. Nat. Commun..

[57] Ahel I (2008). Poly(ADP-ribose)-binding zinc finger motifs in DNA repair/checkpoint proteins. Nature.

[58] Vas AC, Andrews CA, Kirkland Matesky K, Clarke DJ (2007). In vivo analysis of chromosome condensation in Saccharomyces cerevisiae. Mol. Biol. Cell.

[59] Mora-Bermudez F, Gerlich D, Ellenberg J (2007). Maximal chromosome compaction occurs by axial shortening in anaphase and depends on Aurora kinase. Nat. Cell Biol..

[60] Lavoie BD, Hogan E, Koshland D (2004). In vivo requirements for rDNA chromosome condensation reveal two cell-cycle-regulated pathways for mitotic chromosome folding. Genes Dev..

[61] Hirota T, Lipp JJ, Toh BH, Peters JM (2005). Histone H3 serine 10 phosphorylation by Aurora B causes HP1 dissociation from heterochromatin. Nature.

[62] Fischle W (2005). Regulation of HP1-chromatin binding by histone H3 methylation and phosphorylation. Nature.

[63] Wang F (2010). Histone H3 Thr-3 phosphorylation by Haspin positions Aurora B at centromeres in mitosis. Science.

[64] Kelly AE (2010). Survivin reads phosphorylated histone H3 threonine 3 to activate the mitotic kinase Aurora B. Science.

[65] Monaco L (2005). Inhibition of Aurora-B kinase activity by poly(ADP-ribosyl)ation in response to DNA damage. Proc. Natl Acad. Sci. USA.

[66] Slade D (2019). Mitotic functions of poly(ADP-ribose) polymerases. Biochem. Pharmacol..

[67] Halappanavar SS, Shah GM (2004). Defective control of mitotic and post-mitotic checkpoints in poly(ADP-ribose) polymerase-1(−/−) fibroblasts after mitotic spindle disruption. Cell Cycle.

[68] Kanai M (2003). Involvement of poly(ADP-ribose) polymerase 1 and poly(ADP-ribosyl)ation in regulation of centrosome function. Mol. Cell. Biol..

[69] Menissier de Murcia J (2003). Functional interaction between PARP-1 and PARP-2 in chromosome stability and embryonic development in mouse. EMBO J..

[70] Tong WM (2007). Poly(ADP-ribose) polymerase-1 plays a role in suppressing mammary tumourigenesis in mice. Oncogene.

[71] Gemble S (2015). Pyrimidine pool disequilibrium induced by a cytidine deaminase deficiency inhibits PARP-1 activity, leading to the under replication of DNA. PLoS Genet..

