# Supplementary material for: Linking DNA repair and cell cycle progression through serine ADP-ribosylation of histones
Source: Nat Commun. 2022 Jan 13;13:185. doi: 10.1038/s41467-021-27867-4 (PMC8758696; doi:10.1038/s41467-021-27867-4)
Supplement: Supplementary file 1 — Supplementary Information [file 41467_2021_27867_MOESM1_ESM.pdf]

Supplementary Information

Linking DNA repair and cell cycle progression through serine ADP-ribosylation of histones

Julien Brustel, Tetsuya Muramoto, Kazuki Fumimoto, Jessica Ellins, Catherine J. Pears, Nicholas D. Lakin

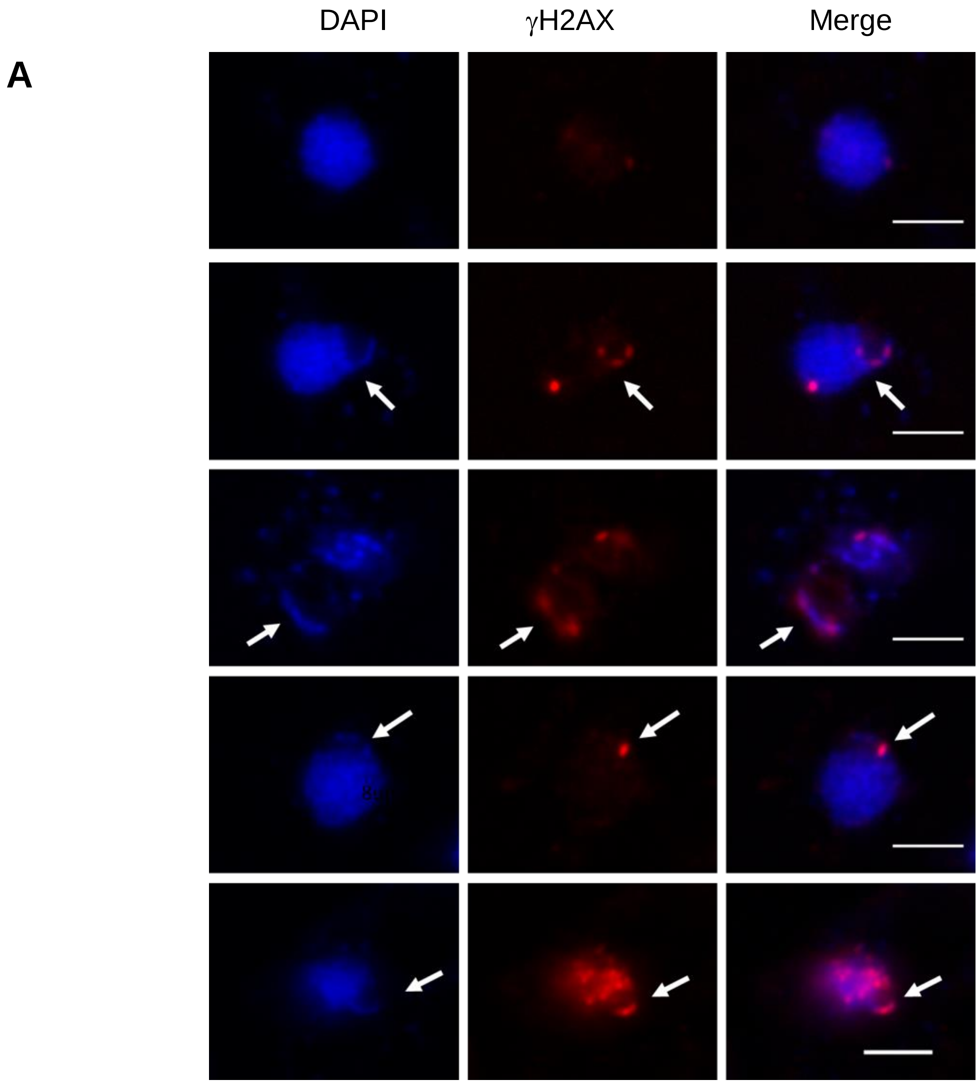

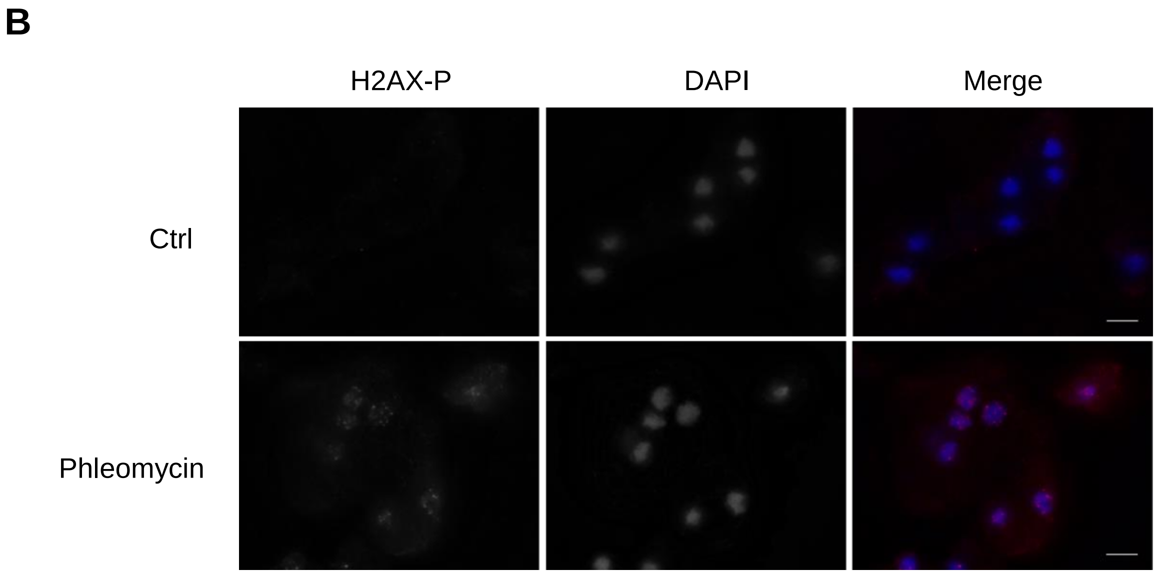

Supplementary Figure 1. Genome instability in *Dictyostelium* cells.

A. Abnormal nuclear morphology of $h3b^{-}$ cells can be associated with DNA damage. Representative immunofluorescence pictures of three independent experiments displaying cells with healthy nuclear (top panel) or abnormal nuclear morphology (bottom four panels) are represented, highlighted with a white arrow. DNA is stained with DAPI (blue) and DNA damage with an antibody against γH2AX (red). Scale bars: 8µm **B.** Ax2 cells were untreated (top panels) or treated (bottom panels) with the DSB inducing drug, phleomycin for 1h. Immunofluorescence with a specific antibody against γH2AX (red), the DNA is stained with DAPI (blue). Scale bars: 10µm. Representative pictures of three independent experiments quantified in Figure 1c.

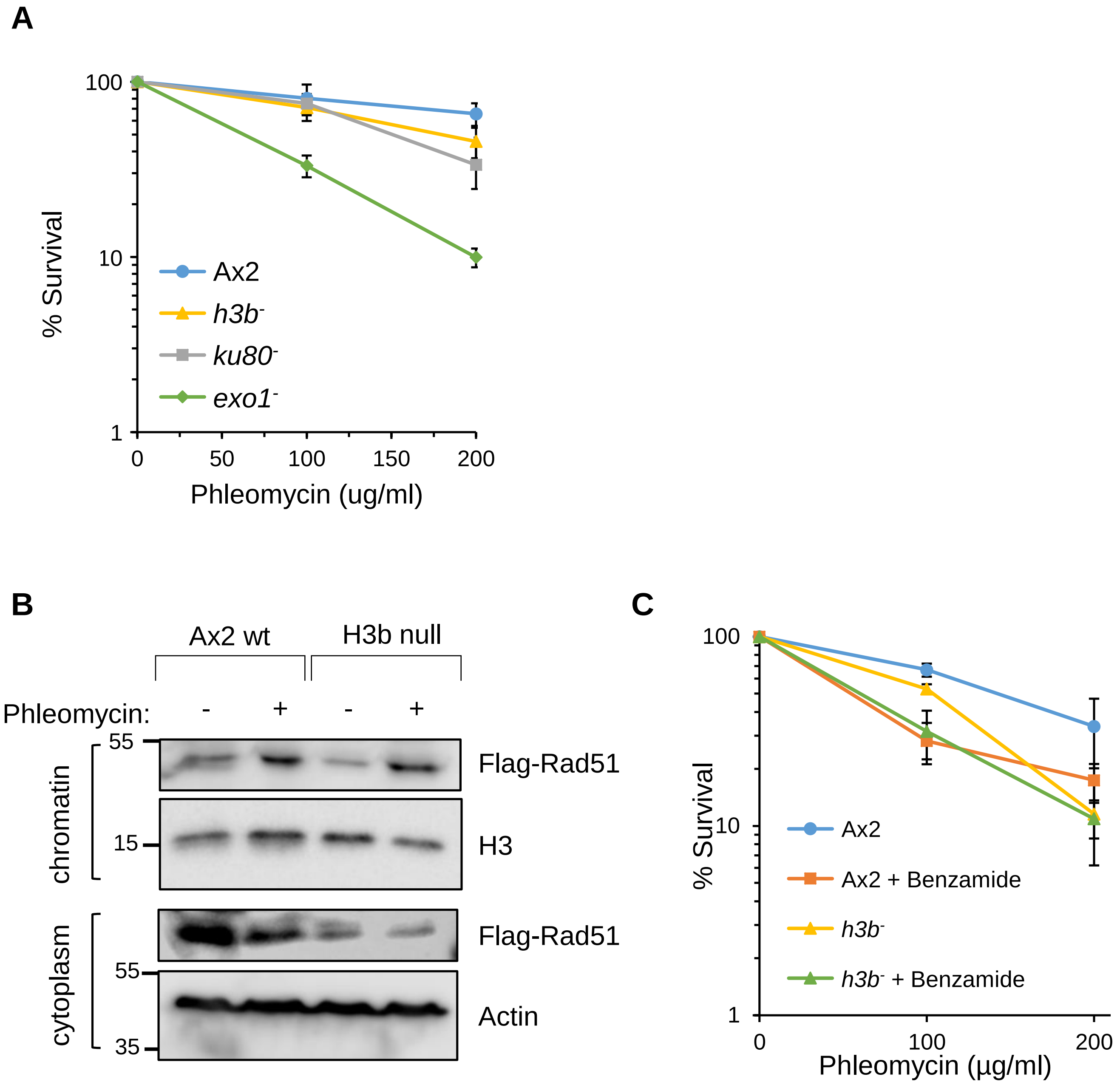

Supplementary Figure 2. Analysis of DNA repair mechanisms in the *h3b*$^{-}$ strain.
A. Sensitivity of different DNA repair mutant strains to phleomycin. Ax2, *h3b*$^{-}$, *exo1*$^{-}$ and *ku80*$^{-}$ cells were exposed to phleomycin for 1h at the indicated concentrations and cell survival assessed by clonogenic survival assays. Data represent 4 biological repeats and error bars represent SEM. **B.** Flag-Rad51 is enriched in chromatin following exposure of the *h3b*$^{-}$ strain to DSBs. Following exposure of Ax2 or *h3b*$^{-}$ cells expressing Flag-Rad51 to phleomycin for 1 hour, cytoplasmic and chromatin fractions were prepared from cells and western blotting performed using the indicated antibodies (representative picture of 2 biological repeats). Molecular weight markers are indicated in kDa. **C.** Sensitivity the *h3b*$^{-}$ strain to phleomycin is epistatic with the PARP inhibitor benzamide. Ax2 or *h3b*$^{-}$ cells were exposed to phleomycin in the absence or presence of benzamide for 1h at the indicated concentrations and cell survival assessed by clonogenic survival assays. Data represent 4 biological repeats and error bars represent SEM. Source data are provided in the Source Data file.

A

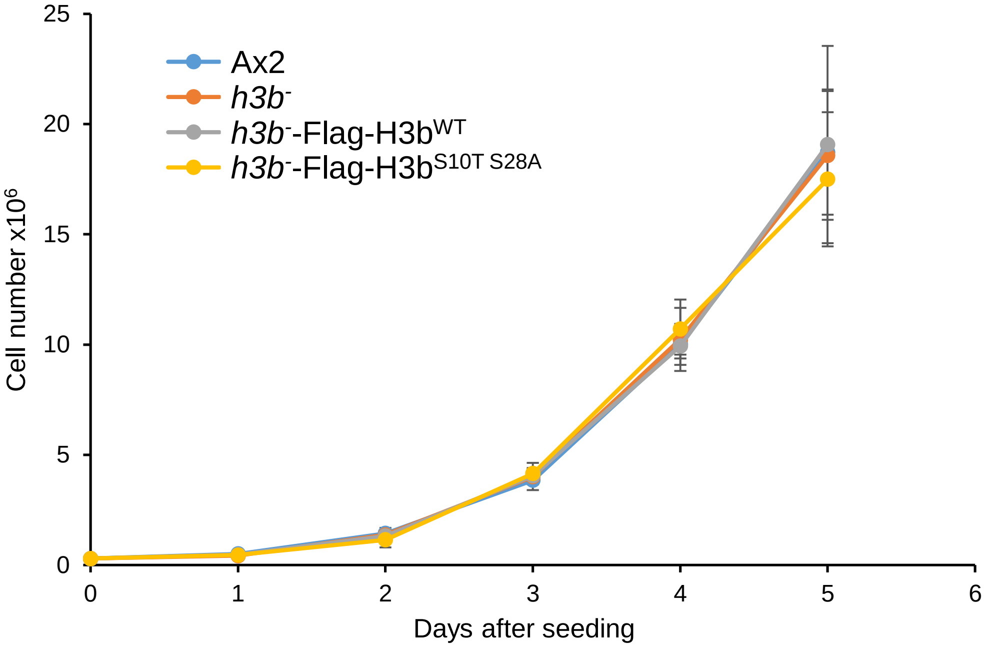

B

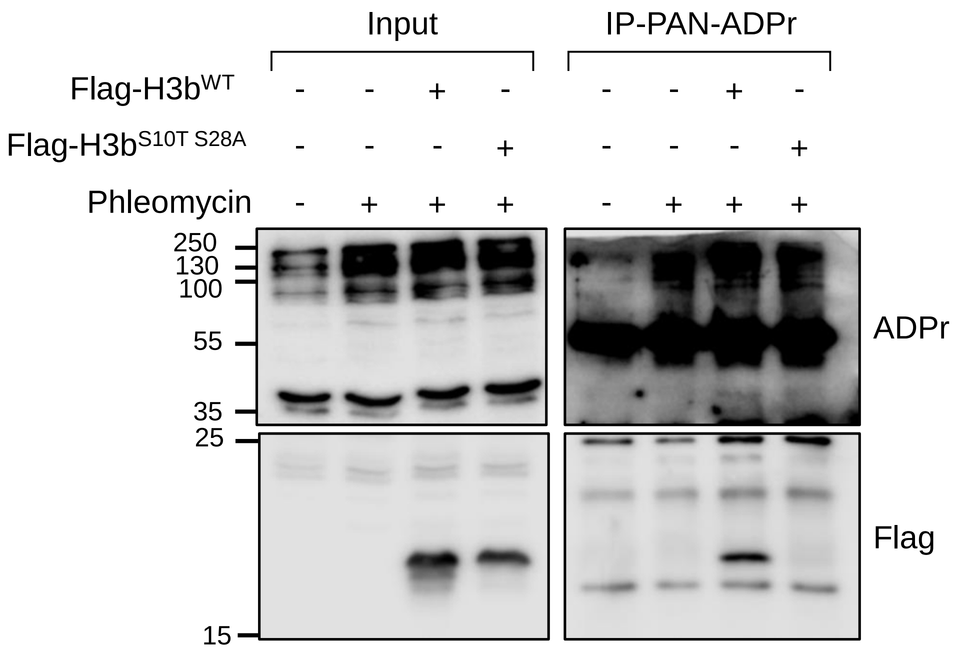

Supplementary Figure 3. Cell growth rates and ADP-ribosylation analysis of the Flag-H3b^{S10TS28A} mutant.

A. Comparison of growth rates of *h3b*$^{-}$ cells expressing Flag-H3b or Flag-H3b^{S10TS28A} mutants. Ax2, *h3b*$^{-}$, or *h3b*$^{-}$ expressing Flag-H3b^{WT} or Flag-H3b$^{S10T\ S28A}$ were seeded at a density of 3x10^{5} cells/ml, and cultivated at 22°C in shaking suspension. Cell number was measured every 24h, for 5 days (data represent 3 biological repeats and error bars represent the SEM). **B.** Flag-H3b, but not Flag-H3b^{S10TS28A} co-purifies with ADP-ribosylated proteins. ADPr proteins were affinity purified from cell extracts using the PAN-ADPr detection reagent. Precipitations were western blotted using the indicated antibodies. Molecular weight markers are indicated in kDa. Representative experiment of 3 biological repeats. Source data are provided in the Source Data file.

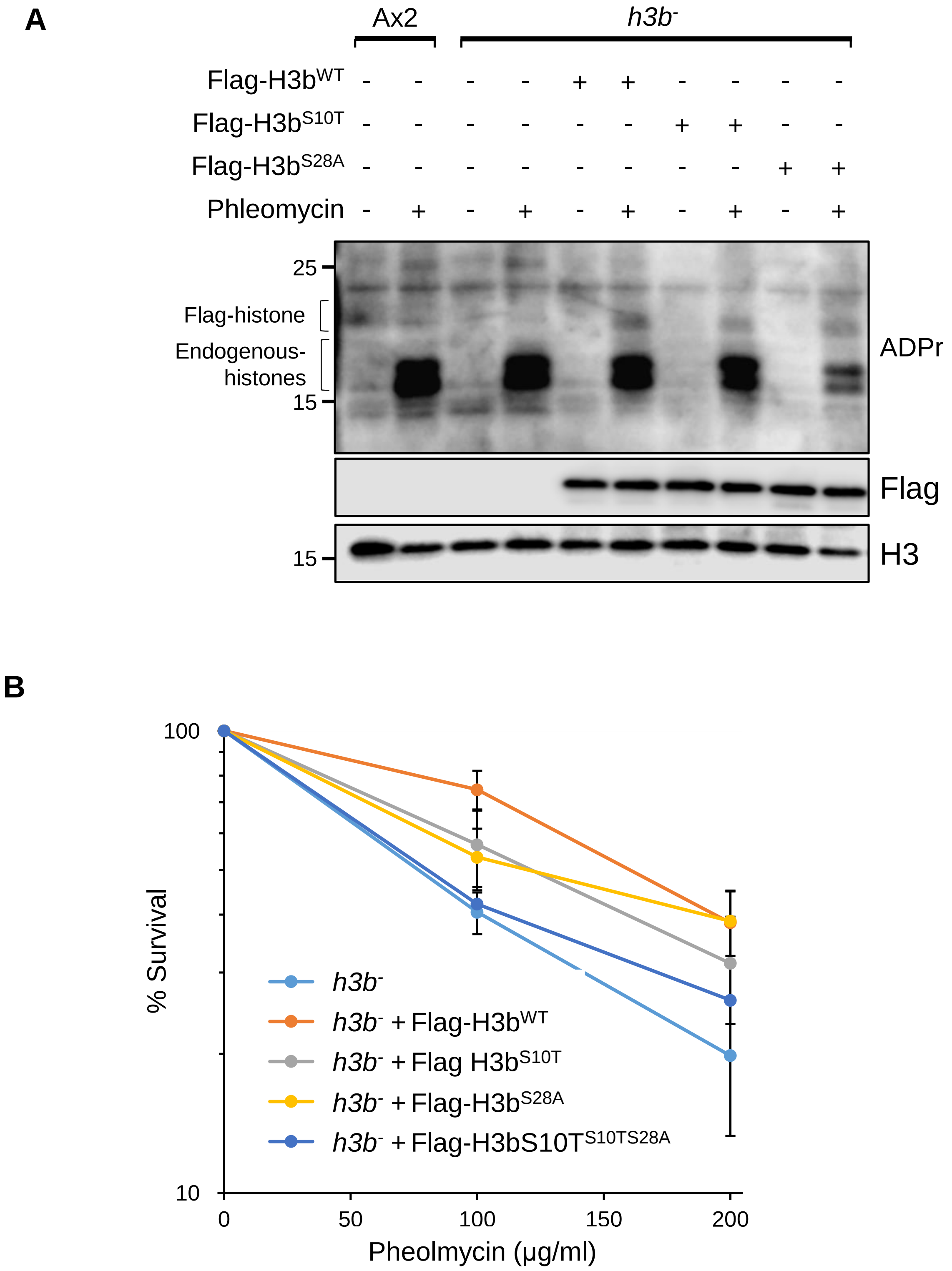

Supplementary Figure 4. Analysis of Flag-H3b^{S10T} and Flag-H3b^{S28A} mutants.
A. Flag-H3b^{S10T} and Flag-H3b^{S28A} are ADP-ribosylated in response to DNA damage. The indicated cells were left untreated or exposed to phleomycin. Following preparation of acid extracts, western blotting was performed using the indicated antibodies. ADP-ribosylated endogenous histones and Flag-H3b are highlighted. Molecular weight markers are indicated in kDa. Representative picture of at least three biological repeats. **B.** Flag-H3b^{S10T} and Flag-H3b^{S28A} do not restore resistance to phleomycin of the *h3b*$^{-}$ strain to the same degree as Flag-H3b^{WT}. The *h3b*$^{-}$ strain alone, or expressing Flag-H3b^{WT}, Flag-H3b^{S10T}, Flag-H3b^{S28A} or Flag-H3b$^{S10T\ S28A}$ was exposed to phleomycin for 1h at the indicated concentrations and cell survival assessed by clonogenic survival assays. Data represent 3 biological repeats, error bar: SEM. Source data are provided in the Source Data file.

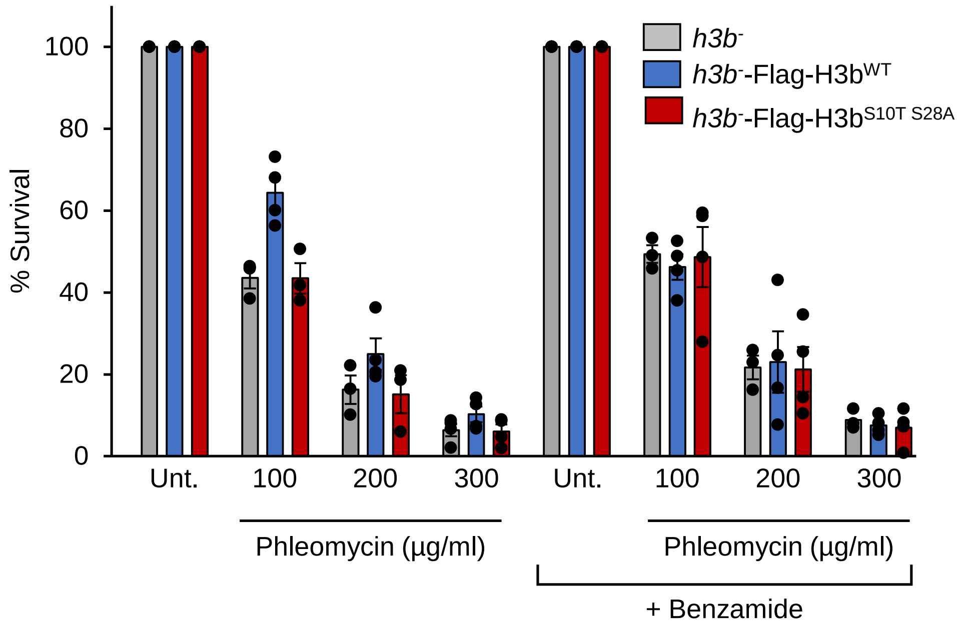

Supplementary Figure 5. Cell survival analysis of *h3b*⁻ strains expressing wild-type or Flag-H3b^{S10TS28A} in the absence or presence of the PARPi benzamide.
The *h3b*⁻ strain alone, or expressing the indictated Flag-tagged H3b mutants were exposed to phleomycin for 1h at the indicated concentrations in the presence or absence of benzamide. Cell survival was assessed by clonogenic survival assays. Data represent 4 biological repeats and error bars represent SEM. Source data are provided in the Source Data file.

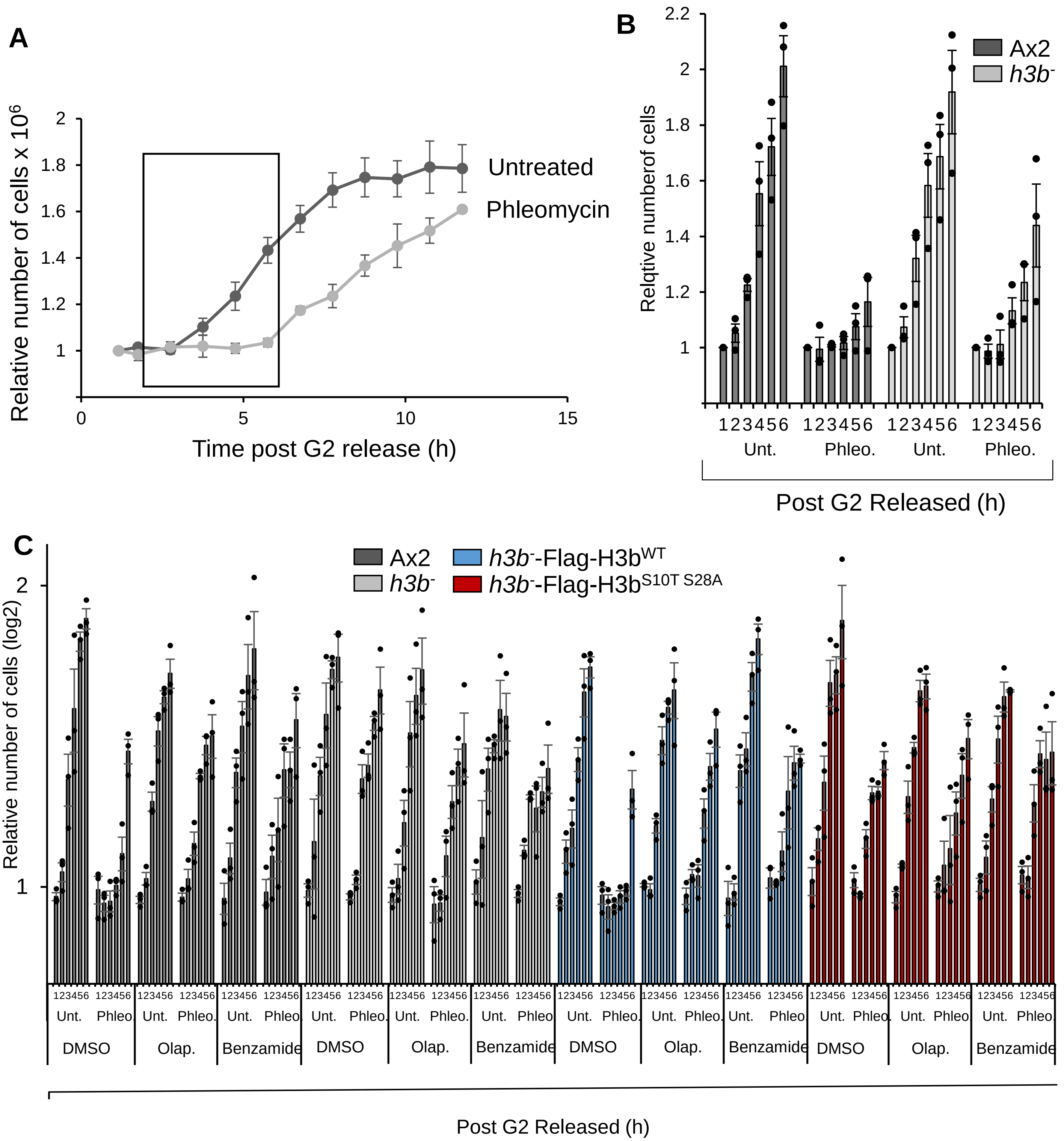

Supplementary Figure 6. ADP-ribosylation of H3b restrains mitotic entry in response to DNA damage.

A. Delay in cell cycle progression following exposure of cells to phleomycin. The *h3b*$^-$ strain expressing Flag-H3b^{WT} was arrested in G2 and following treatment or not with phleomycin, released into fresh media to allow resumption of the cell cycle. Progression through mitosis was monitored by assessing cell number at times following release from G2. The black box represents the time points displayed in Figure 5. Data represent 3 biological repeats and error bars represent the SEM. **B.** The *h3b*$^-$ strain enters mitosis prematurely following exposure of cell to phleomycin. Ax2 or *h3b*$^-$ were exposed to phleomycin and following release into fresh media, entry into mitosis analysed as in (A). Data represent 3 biological repeats and error bars represent the SEM. **C.** Premature mitotic entry of ADP-ribosylation mutants following exposure to DNA damage is epistatic with the PARP inhibitors olaparib and benzamide. Ax2, *h3b*$^-$ cells, or *h3b*$^-$ cells expressing wild-type or Flag-H3b^{S10TS28A} were exposed to phleomycin and following release into fresh media, entry into mitosis analysed as in (A) (data represent 3 biological repeats, error bar: SEM). DMSO and Olaparib samples are from the same data set displayed in the main Figure 5b. Source data are provided in the Source Data file.

A

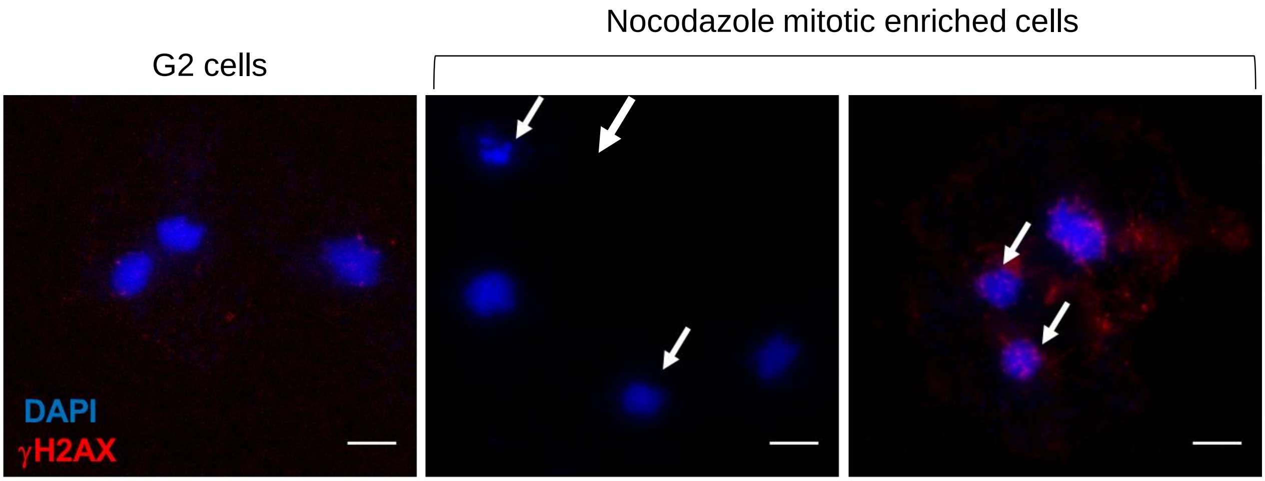

B

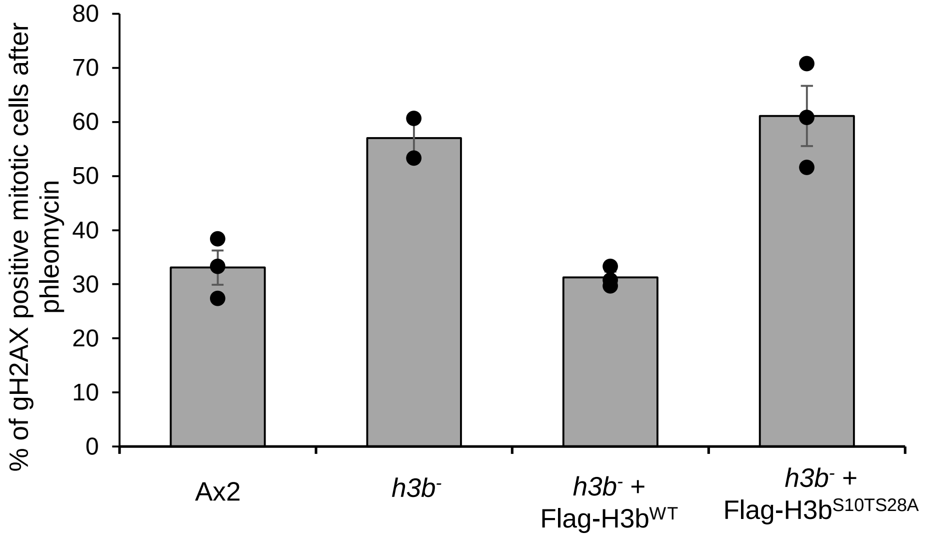

Supplementary Figure 7. Flag-H3b^{S10TS28A} mutants enter mitosis prematurely with DNA damage

Ax2 or *h3b*$^{-}$ cells expressing Flag-H3b^{WT} or Flag-H3b$^{S10T\ S28A}$ were arrested in G2 and following treatment or not with phleomycin, released into fresh media to allow resumption of the cell cycle, and in presence of nocodazole to enrich for mitotic population. 6 to 8 hours after release, cells were fixed and DNA damage assessed by assessing γH2AX levels in mitotic cells by immunofluorescence. (A) Representative immunofluorescence pictures of mitotic cells without (middle panel, white arrows) or with (right panel, white arrows) unrepaired DNA damage. Scale bars: 5µm (B) Quantification of γH2AX-positive mitotic cells. Representative pictures and quantification of 3 biological repeats, error bar SEM. Source data are provided as a Source Data file.
