# Supplementary material for: Linking DNA repair and cell cycle progression through serine ADP-ribosylation of histones
Source: Nat Commun. 2022 Jan 13;13:185. doi: 10.1038/s41467-021-27867-4 (PMC8758696; doi:10.1038/s41467-021-27867-4)
Supplement: Supplementary file 2 — Description of additional Supplementary File [file 41467_2021_27867_MOESM2_ESM.pdf]

Descriptions for additional supplementary data files

Supplementary Movie 1: Time-lapse movie showing representative anaphase bridges during cytokinesis. Time-lapse images were acquired every 30 seconds. Scale bar: 10 µm

Supplementary Movie 2 Time-lapse movie showing representative micronuclei. Timelapse images were acquired every 30 seconds. Scale bar: 5 µm.
